# Spatial Lipidomic Profiling of Mouse Joint Tissue Demonstrates the Essential Role of PHOSPHO1 in Growth Plate Homeostasis

**DOI:** 10.1002/jbmr.4796

**Published:** 2023-03-14

**Authors:** Jordan Tzvetkov, Louise A. Stephen, Scott Dillon, Jose Luis Millan, Anke J. Roelofs, Cosimo De Bari, Colin Farquharson, Tony Larson, Paul Genever

**Affiliations:** ^1^ York Biomedical Research Institute and Department of Biology University of York York UK; ^2^ The Roslin Institute University of Edinburgh Edinburgh UK; ^3^ Wellcome‐Medical Research Council (MRC) Cambridge Stem Cell Institute University of Cambridge Cambridge UK; ^4^ Sanford Burnham Prebys, Medical Discovery Institute La Jolla CA USA; ^5^ Centre for Arthritis and Musculoskeletal Health University of Aberdeen Aberdeen UK

**Keywords:** GROWTH PLATE, MATRIX MINERALIZATION, BONE MODELING AND REMODELING, STATISTICAL METHODS, DISORDERS OF CALCIUM/PHOSPHATE METABOLISM

## Abstract

Lipids play a crucial role in signaling and metabolism, regulating the development and maintenance of the skeleton. Membrane lipids have been hypothesized to act as intermediates upstream of orphan phosphatase 1 (PHOSPHO1), a major contributor to phosphate generation required for bone mineralization. Here, we spatially resolve the lipid atlas of the healthy mouse knee and demonstrate the effects of PHOSPHO1 ablation on the growth plate lipidome. Lipids spanning 17 subclasses were mapped across the knee joints of healthy juvenile and adult mice using matrix‐assisted laser desorption ionization imaging mass spectrometry (MALDI‐IMS), with annotation supported by shotgun lipidomics. Multivariate analysis identified 96 and 80 lipid ions with differential abundances across joint tissues in juvenile and adult mice, respectively. In both ages, marrow was enriched in phospholipid platelet activating factors (PAFs) and related metabolites, cortical bone had a low lipid content, whereas lysophospholipids were strikingly enriched in the growth plate, an active site of mineralization and PHOSPHO1 activity. Spatially‐resolved profiling of PHOSPHO1‐knockout (KO) mice across the resting, proliferating, and hypertrophic growth plate zones revealed 272, 306, and 296 significantly upregulated, and 155, 220, and 190 significantly downregulated features, respectively, relative to wild‐type (WT) controls. Of note, phosphatidylcholine, lysophosphatidylcholine, sphingomyelin, lysophosphatidylethanolamine, and phosphatidylethanolamine derived lipid ions were upregulated in PHOSPHO1‐KO versus WT. Our imaging pipeline has established a spatially‐resolved lipid signature of joint tissues and has demonstrated that PHOSPHO1 ablation significantly alters the growth plate lipidome, highlighting an essential role of the PHOSPHO1‐mediated membrane phospholipid metabolism in lipid and bone homeostasis. © 2023 The Authors. *Journal of Bone and Mineral Research* published by Wiley Periodicals LLC on behalf of American Society for Bone and Mineral Research (ASBMR).

## Introduction

Lipid signaling and membrane lipid composition play an important role in skeletal development and maintenance. Reduced lipid availability in avascular cartilage drives skeletal progenitor cell commitment to the chondrogenic lineage in a SOX9‐dependent manner.^(^ ^1^ ^)^ Lipids have also been implicated in the Wnt‐mediated control of osteoclast/osteoblast function,^(^ ^2^, ^3^, ^4^, ^5^ ^)^ whereas vitamin D has been extensively studied as a regulator of bone growth, homeostasis, and health.^(^ ^6^, ^7^ ^)^ Lipid imbalances have been implicated in multiple bone and joint pathologies, with various skeletal tissues displaying altered lipid profiles in different disease states.^(^ ^8^ ^)^ To date, most lipid‐based studies in bone have used shotgun lipidomics on lipid extracts, which catalogues the underlying lipid species, but lacks the spatial resolution necessary to map lipids from specific cell and tissue types to precise anatomical locations. Spatially‐resolved analyses can also reduce the inherent measurement bias of a pooled extract toward ubiquitously abundant molecules, enabling the detection of specific compounds in cell subpopulations with niche distributions that may play a critical role in tissue homeostasis.

Imaging mass spectrometry (IMS) has emerged as a powerful tool for biomolecular fingerprinting of tissues in a spatially‐resolved manner. Since its first use to map proteins and peptides across the rat spleen and pancreas,^(^ ^10^ ^)^ matrix‐assisted laser desorption ionization (MALDI)‐IMS has been particularly useful in the study of lipid biochemistry and in determining the function of these important signaling metabolites outside of their traditional role as membrane structural components and energy storage molecules.^(^ ^11^ ^)^ It has, for example, led to the discovery of lipid markers that differentiate between stromal and tumor microenvironments,^(^ ^12^, ^13^, ^14^, ^15^ ^)^ and helped to elucidate the metabolic changes associated with pathologies such as Alzheimer's and Huntington's disease.^(^ ^16^, ^17^, ^18^, ^19^ ^)^ MALDI‐IMS–driven lipidomics have also been central to both targeted and untargeted discoveries of lipid markers associated with pathophysiology, normal biochemical function, and metabolism across a wide range of model organisms and human tissues, with liquid chromatography tandem mass spectrometry (LC‐MS/MS)‐coupled approaches proving especially useful in lipid identification.^(^ ^19^, ^20^, ^21^, ^22^, ^23^ ^)^

Despite this, MALDI‐IMS approaches have seen limited application in bone primarily due to the challenging nature of this tissue; hydroxyapatite can hinder the identification of biomolecule distribution patterns.^(^ ^24^ ^)^ However, proof‐of‐concept method development studies have demonstrated the feasibility of applying MALDI‐IMS and similar techniques to bone and cartilage tissues,^(^ ^25^, ^26^, ^27^, ^28^ ^)^ with recent advances enabling the biochemical fingerprinting of undecalcified tissues^(^ ^29^, ^30^ ^)^ and mapping the lipidomic changes in osteoarthritis (OA) synovial tissues.^(^ ^31^ ^)^ However, our knowledge of the biological relevance of lipid distributions across bone and joint tissues remains limited. A key skeletal development process in which lipids have been implicated, but not spatially resolved, is bone mineralization. During mineralization, matrix vesicles (MVs) are secreted by chondrocytes, odontoblasts and osteoblasts to initiate mineralization in cartilage, dentin and bone, respectively. MVs, first discovered in growth plate cartilage, are small membrane bound vesicles (100–300 nm in diameter) that are rich in lipids and proteins known to concentrate calcium and inorganic phosphate (P_i_) ions and are responsible for the initiation of matrix mineralization.^(^ ^32^, ^33^ ^)^ The P_i_ pool required for mineralization is accumulated under the action of phosphatases.^(^ ^34^ ^)^ Tissue‐nonspecific alkaline phosphatase (TNAP) has long been established to play a central role in the mineralization of the extracellular matrix (ECM).^(^ ^35^, ^36^ ^)^ Deletion of *Alpl* (the gene encoding TNAP) in mice causes a reduction in but not a complete abolition of mineralization of the skeleton, as evidenced by the presence of hydroxyapatite crystals within MVs and the ECM of bones from *Alpl* null mice.^(^ ^37^ ^)^ The recognition for the requirement of other phosphatases for ECM mineralization led to the identification of orphan phosphatase 1 (PHOSPHO1) as a pivotal regulator of bone mineralization during bone development and fracture repair, as demonstrated by both in vivo and cell culture studies.^(^ ^38^, ^39^, ^40^, ^41^, ^42^, ^43^, ^44^, ^45^, ^46^, ^47^ ^)^

PHOSPHO1 is specifically expressed at mineralization sites, including the hypertrophic zone of the growth plate, as shown in both chicks and mice.^(^ ^40^, ^48^, ^49^ ^)^ Similar to the TNAP‐null mouse, PHOSPHO1‐knockout (KO) mice exhibit a hypomineralized phenotype.^(^ ^42^ ^)^ Yadav and colleagues^(^ ^42^ ^)^ observed that PHOSPHO1‐KO bones are bowed and present with spontaneous greenstick fractures at birth, which was attributed to an inability of the hypomineralized bone to withstand physiological loading. This hypomineralized phenotype is likely to stem from a reduced availability of P_i_ to form hydroxyapatite as a result of PHOSPHO1 ablation.^(^ ^38^, ^42^ ^)^ PHOSPHO1 exhibits specificity for the membrane phospholipid headgroups phosphocholine (PCho) and phosphoethanolamine (PEtOH),^(^ ^50^ ^)^ but the upstream pathways for the generation of these substrates remain largely unknown. It has been proposed that MV membrane remodeling under the action of an unidentified phospholipase A2 (PLA2) enzyme converts phosphatidylcholine (PC) lipids to a lyso‐phosphatidylcholine (LPC) lipid intermediate, which is subsequently hydrolyzed by ectonucleotide pyrophosphatase/phosphodiesterase 6 (ENPP6) to generate the PCho substrate for PHOSPHO1.^(^ ^51^ ^)^ The involvement of ENPP6 as an upstream mediator of PHOSPHO1 function was recently supported by the hypomineralized phenotype observed in ENPP6‐null mice.^(^ ^52^ ^)^ Nevertheless, the precise role of PHOSPHO1 in lipid homeostasis and metabolism during bone mineralization in the growth plate is yet to be elucidated.

In this study, we used mouse knee joint tissues to develop a pipeline for MALDI‐IMS data acquisition and analysis. For the first time, we generated a lipid atlas of the juvenile and adult mouse knee joints and identified a biomolecular lipid fingerprint for articular cartilage, growth plate cartilage, bone marrow, and cortical bone. In applying this analytical pipeline to PHOSPHO1‐KO joint tissue, we were able to demonstrate the prominent role of PHOSPHO1 in lipid homeostasis and provide supporting evidence for unresolved PHOSPHO1 biochemical pathways.

## Materials and Methods

### Tissue handling

The use of mouse tissues during pipeline development was approved by the University of York Animal Welfare Ethical Review Body. The PHOSPHO1‐KO mice were generated as described.^(^ ^41^ ^)^ All animal experiments were approved by the Roslin Institute's named veterinary surgeon and named animal care and welfare officer (NACWO), with animals maintained in accordance with the Home Office code of practice (for the housing and care of animals bred, supplied, Animals in Research: Reporting In Vivo Experiments [ARRIVE] guidelines or used for scientific purposes).

Whole mouse knee joints were obtained from juvenile (3–4 weeks old) (*n* = 3) and adult (12 weeks old) (*n* = 3) male C57BL/6 mice, and male juvenile PHOSPHO1‐KO mice (*n* = 4) and wild‐type (WT) control mice on the same genetic background as the PHOSPHO1‐KO mice (*n* = 4). Upon harvesting the mouse limbs, the skin was removed and tissues were immediately fixed and decalcified in 10% trichloroacetic acid (TCA, Sigma‐Aldrich, Gillingham, UK) solution at 4°C for 24 hours. Tissues were subsequently rinsed in 1× PBS (Oxoid Limited, Basingstoke, UK) and cryoprotected in 30% sucrose (Thermo Fisher Scientific, Loughborough, UK)/1× PBS with shaking. Finally, tissues were embedded in 3% carboxymethylcellulose (CMC, ultra high viscosity, highly purified, Sigma‐Aldrich, Gillingham, UK) in a plastic mold using a dry ice/ethanol (EtOH) slurry and stored at −70°C prior to sectioning.

### Cryosectioning

The embedded tissue blocks were mounted on brass chucks using 3% CMC. Knee joints were sectioned at specimen temperature −20°C and chamber temperature −22°C using a cryostat (model OTF5000; Bright Instruments Ltd, Huntingdon, UK) equipped with a tungsten carbide blade. Serial tissue sections of 12 μm thickness were collected on Thermo Scientific™ SuperFrost adhesion slides (Thermo Fisher Scientific, Loughborough, UK) and left to dry at room temperature for 3 hours to facilitate adhesion. The sections were inspected for morphological integrity under a microscope; the highest quality representative sections were selected for MALDI‐IMS data acquisition and optically scanned at 4800 dots per inch (dpi), equivalent to 5.29 × 5.29 μm raster pixels using an EPSON PERFECTION 4990 PHOTO scanner. Tissue sections were then stored at −70°C in a glass holder box with desiccant prior to matrix application.

### Safranin‐O histological staining

For histological staining following MALDI‐IMS, the matrix was removed by immersing the sections slides for 1 minute each in serial dilutions of ice cold 70%, 90%, and finally 100% EtOH (Thermo Fisher Scientific, Loughborough, UK). Sections were counterstained in 0.1% Fast Green FCF (Thermo Fisher Scientific, Loughborough, UK) for 5 minutes, washed in 1% acetic acid (Thermo Fisher Scientific, Loughborough, UK) for 30 seconds, stained in 0.1% Safranin O (Sigma‐Aldrich, Gillingham, UK) for 15 minutes, washed in distilled water (dH_2_O) for 1 minute, and dehydrated in 70%, 90%, and 100% EtOH with 10 dips in each ascending concentration and a final step of 5 minutes in xylene (Thermo Fisher Scientific, Loughborough, UK). The sections were air dried, mounted with dibutylphthalate polystyrene xylene mounting medium (CellPath, Newtown, UK) and scanned in Positive Film at 4800 dpi using an EPSON PERFECTION 4990 PHOTO scanner. Parallel sections were stained and imaged under a Leica DM2000 LED microscope using a Leica DC 500 camera and DFC500Twain v7.5.1 software (Leica, Wetzlar, Germany).

### LC‐MS/MS

#### Data acquisition

Juvenile mouse joint tissues were fixed and decalcified as described in the Tissue Handling section, but the muscle tissues were removed. The whole joints were wrapped in aluminum foil, flash‐frozen in liquid nitrogen and mechanically ground to a powder. Of the resulting powder, 150–200 mg from each sample was transferred to a glass vial for lipid extraction. HPLC‐grade dichloromethane/methanol (both purchased from Thermo Fisher Scientific, Loughborough, UK) solvent was prepared at 2:1 vol/vol ratio and 2 mL was added to each glass vial. Samples were sonicated in a water bath and subsequently centrifuged at 500 *g* for 5 minutes. The supernatant was transferred to a new glass vial. The extraction process was repeated three times, and the pooled supernatant was subsequently lyophilized in a vacuum drier until a dry lipid pellet was formed, which was stored at −70°C. The lipid pellet was resolubilized in 70:30 MeCN:iPrOH (both purchased from Thermo Fisher Scientific, Loughborough, UK) and LC‐MS/MS was performed using 2 μL injection volume in separate positive and negative mode acquisitions using an Orbitrap mass spectrometer (Thermo Fisher Scientific, Loughborough, UK), as described.^(^ ^52^ ^)^

#### Data processing

Data processing and downstream analysis were performed in R, using Bioconductor package XCMS^(^ ^54^ ^)^ and processed as described.^(^ ^53^ ^)^ MS1 spectra were matched against the LipidMaps Structural Database,^(^ ^55^ ^)^ and associated MS2 spectra were searched against the Lipid Match^(^ ^56^ ^)^ and Lipid Blast databases.^(^ ^57^ ^)^ To generate consensus m/z‐only values for MALDI‐MSI m/z annotation, retention time data was removed and xcms‐derived LCMS MS1 m/z values binned into 0.2 m/z width bins, as described.^(^ ^58^ ^)^

### MALDI‐IMS

#### Matrix application

In all experiments 2,5‐dihydroxybenzoic acid (DHB, Sigma‐Aldrich, Gillingham, UK) matrix solution was freshly prepared at 20 mg/mL in 0.1% trifluoroacetic acid (Thermo Fisher Scientific, Loughborough, UK) in HPLC grade MeOH/H2O (50/50, vol/vol). For PHOSPHO1‐KO versus WT data acquisition, an internal standard of SPLASH LipidoMix™ purchased from Avanti Polar Lipids (Alabaster, AL, USA) was added to fresh matrix solution at a 1:50 dilution. This standard was selected due to its coverage of a wide variety of lipid classes. The matrix was subsequently applied evenly across the tissue section slides using an HTX automated sprayer system (HTX Imaging, Chapel Hill, NC, USA). The following settings were used: 80°C spray nozzle temperature, 12 criss‐cross passes and offset, 50 μL/min flow rate, 1250 mm/min spray nozzle velocity, 3 mm track spacing, 10 psi Nitrogen pressure, 3 L/min gas flow rate, 10 seconds drying time, and nozzle height 40 mm.

#### Data acquisition

MALDI‐IMS data was acquired in positive ionization mode on a Waters Synapt‐G2‐Si mass spectrometer (Waters Corp., Milford, MA, USA). The instrument was calibrated using red phosphorus, with regularly spaced cluster ions across the mass range to achieve a root mean square (RMS) mass error of ≤10 parts per million (ppm). MS1 spectra were acquired in sensitivity mode over the entire cross‐section of the joint, with the acquisition area defined by Waters™ High Definition Imaging (HDI) version 1.5 software (Waters Corp.). Ablation pixel size was set to 50 × 50 μm. Scan time was 0.5 seconds, laser rate – 1 kHz, and the m/z range was 100–2000. Ion mobility separation was enabled for the separation of isobaric molecules during pipeline development and in the adult versus juvenile data acquisition, but disabled in the PHOSPHO1‐KO versus WT data acquisition to improve sensitivity. Waters™ MajorMix (Waters Corp.) was used for collision cross section (CCS) calibrations for ion mobility.

#### Data processing

##### For pipeline development

The acquired data was initially processed in HDI to include the top 3000 most intense ion peaks with intensity >100 for noise removal. The HDI target list building function was used on the resulting processed files to generate a list of detected ions, with the m/z bin window set to 0.04 and drift time bin window set to 100, resulting in a list of 4350 targets. This target list was used to re‐process all raw files, splitting the ion intensity data into bins with the same labels across all samples and allowing for subsequent comparison between samples.

##### For PHOSPHO1‐KO versus WT analysis

The acquired data was initially processed in HDI to include the top 3000 most intense ion peaks with intensity >100. To ensure ppm mass error was minimized across samples, lock mass correction was enabled during this step using the most intense internal standard ion detected at m/z 753.6134, which was the [M + H]^+^ ion for deuterated PC (15:0–18:1(d7)), added from the SPLASH LipidoMix™. To enable between‐groups comparison, the generated files were further processed through the BASIS normalization pipeline for peak alignment, intrasample normalization, intersample normalization, and variance scaling.^(^ ^58^ ^)^ The BASIS pipeline aligns and normalizes image data arrays, but retains the original m/z and drift time values in its outputs. These output files were therefore reprocessed using an in‐house R script to calculate mean m/z and drift time values, which were used to update these values in each .txt file used by HDI for subsequent data visualization and region of interest (ROI) export.^(^ ^59^ ^)^

### Lipid ion annotation

The LC‐MS/MS–processed lipid annotations and their respective theoretical MALDI‐generated ions were used as a library for MALDI‐IMS ion annotation. Features detected in the MALDI‐IMS experiments were matched to the theoretical m/z of lipids identified in the LC‐MS/MS experiment using an in‐house R script. Matches were retained within an m/z tolerance of 100 ppm. This tolerance was selected to account for the maximum likely mass error caused by MALDI‐IMS calibration drifts and potential artifacts of binning processes across multi‐day and multi‐month acquisitions. The matching algorithm accounted for the possibility of the MALDI ions being protonated, sodiated, potassiated, and dehydrated adduct forms, as well as C13 isotope peaks.

Positive ion mode LC‐MS/MS features were matched to negative mode features based on retention time, and the MS2 spectra were manually inspected for presence of diagnostic peaks and annotated as described in Fig. S5.

For putative annotation ID hits were subsequently scored as follows: (i) manually annotated lipid ID; (ii) putative ID of LipidMatch and LipidBlast database overlaps; (iii) a highly scored LipidMatch ID but no LipidBlast ID; and (iv) low confidence ID to be excluded based on one or multiple of the following indicators: low LipidMatch score; odd number of carbon atoms present in FA chain; both LC‐MS/MS and MALDI‐IMS peaks identified as an adduct other than protonated, eg, because the hit matches the mass of a doubly sodiated ion in MALDI, but only singly charged ions are expected.

### Lipid nomenclature and notation

LIPID MAPS® nomenclature and notation was used in this publication.^(^ ^61^, ^62^ ^)^ Briefly, the notation follows the following structure: HeadGroup(Sn1AcylGroup/Sn2AcylGroup). For example, *PC* (18:0/18:3) is a *phosphatidylcholine (PC)* with an 18‐carbon long acyl group in both the Sn1 (left of the “/”) and Sn2 (right of the “/”) positions. The Sn1 acyl group is saturated (no double bonds as indicated by the 0 after “:”), whereas the Sn2 acyl group is unsaturated with three double bonds. Where the specific Sn1 and Sn2 acyl groups could not be established, the shorthand notation is used, which follows the structure: HeadGroup(TotalNumberOfCarbonAtomsAcrossAllAcylGroups:TotalNumberOfDoubleBondsAcrossAllAcylGroups).

### Data analysis

#### Data visualization

MALDI‐IMS data were visualized in HDI, where the histologically stained section scans were overlaid with the ion distribution data, enabling accurate tissue‐specific selection of ROIs. Total ion count (TIC) normalization and the hot‐metal color palette were used.

#### ROI selection

During pipeline development, a minimum of three ROIs comprising of 10 ablation pixels each (lROIs) were selected from both the tibia and the femur for each of the following tissues: articular and growth plate cartilage, cortical bone, subchondral bone, and epiphyseal and diaphyseal marrow. Subchondral bone and epiphyseal marrow lROIs comprised 5 pixels instead due to their small surface area. In addition, a minimum of 10 ROIs consisting of single ablation pixels (sROIs) were selected for each morphological feature to assess within tissue variation. For PHOSPHO1‐KO versus WT analysis of the growth plate, a minimum of three 10‐pixel large ROIs were selected from each bone's (tibia and femur) hypertrophic, proliferating, and resting zones of the growth plate.

#### Data processing for multivariate analysis

ROI data was exported for multivariate analysis in HDI and subsequently noise filtered to robustly remove matrix ions using an in‐house R script. Briefly, ROIs outside the tissue were selected, containing DHB matrix only. The mean intensity of each ion in the matrix‐only ROIs was compared to the mean intensity of these ions within all selected tissue‐ROIs, and ions for which (Matrix ROIs mean intensity − 2SD) > (Tissue ROIs mean intensity + 2SD) were excluded. Data were log‐scaled and TIC normalized. Metadata were then merged with the final dataset to enable analysis in EZinfo by Umetrics® (Sartorius, Göttingen, Germany).

#### Multivariate statistical analysis

Principal component analysis (PCA) was carried out in EZinfo using Pareto scaling as a tool for dimensionality reduction of the data—from thousands of detected ions to a few principal components that best summarize the variation in the data. It was applied to visualize group differences in an unsupervised manner. Subsequently, Orthogonal Projections to Latent Structures Discriminant Analysis (OPLS‐DA) was carried out in EZinfo using Pareto scaling to extract differential ions between the groups that were being compared. OPLS‐DA is similar to PCA in its dimensionality reduction, but assumes differences exist between the groups of data being compared. The resulting OPLS‐DA S‐plots were used to robustly select the ions most highly associated with each tissue based on an absolute covariance threshold set to ≥0.04. False positives were discarded after visual inspection of ion distribution, resulting in a final panel of differential ions used in the hierarchical clustering analysis. Hierarchical clustering was performed on z‐score normalized ion intensity data in RStudio.

For the PHOSPHO1‐KO versus WT zone‐specific analyses, a multiple *t* test was carried out in RStudio using package rstatix.^(^ ^63^ ^)^ All MVA plots and diagrams were generated in RStudio using ggplot2.^(^ ^64^ ^)^ Venn diagrams were generated using VennDiagram.^(^ ^65^ ^)^ Heatmaps in Fig. S1 *B* were generated using package pheatmap.^(^ ^66^ ^)^

## Results

### Establishing the MALDI‐IMS spatial lipidomic pipeline

**Fig. 1:**
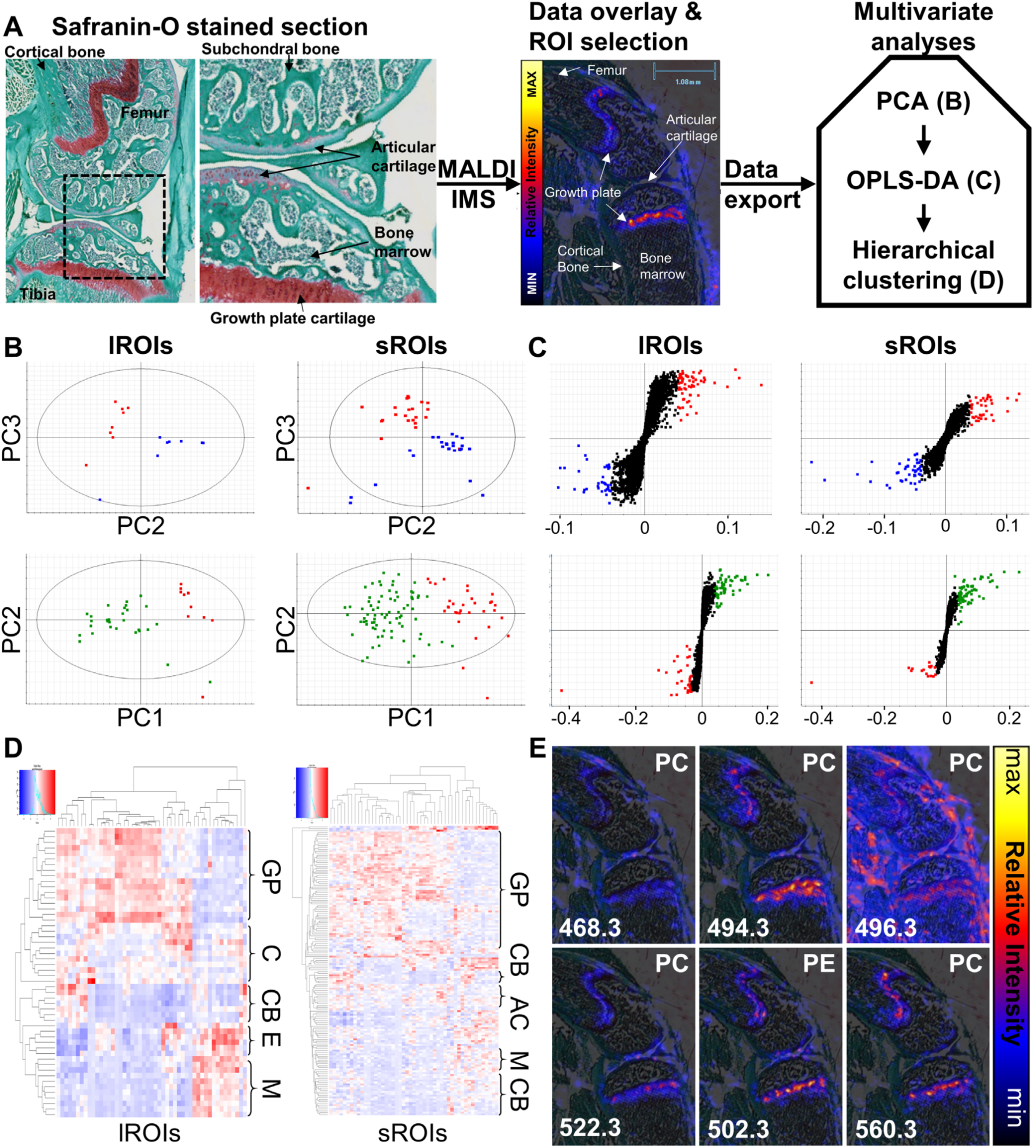
MALDI‐IMS spatial lipidomics pipeline development identifies lysophospholipids in the growth plate. (*A*) Workflow schematic: sections were stained with fast green/Safranin‐O to identify histological features. Following MALDI‐IMS, ion intensity data was overlaid with a histology scan for ROI selection. Single ablation pixel regions of interest (sROIs) and large, 10‐pixel ROIs (lROIs) spanning the diaphyseal and epiphyseal bone marrow, articular and growth plate cartilage, and cortical and subchondral bone were analyzed using multivariate modeling. Representative plots from the analysis in a single biological replicate are shown, with color‐coded bone (blue), cartilage (green), and bone marrow (red). (*B*) PCA scores plots where each point represents an ROI. Plotted are the principal components demonstrating the best separation between tissues. From left to right and top to bottom these were: PC2 (16.5% variance) versus PC3 (12.6% variance); PC2 (9.5% variance) versus PC3 (6.3% variance); PC1 (32.5% variance) versus PC2 (14.8% variance); PC1 (14.4% variance) versus PC2 (4.9% variance). (*C*) OPLS‐DA S‐plots of covariance (w*) (*x*‐axis) against correlation (*y*‐axis). Each point represents an ion. Color‐coded are the ions highly enriched (|w*| > 0.04) in each tissue. (*D*) Hierarchical clustering analysis heat maps with ions in columns and ROIs in rows. Tissue‐specific clusters of ROIs have been labeled to the right. The heat map colors represent z‐score normalized ion intensities, with relative abundance color‐coded as low = cold and high = hot. (*E*) Each image maps the distribution of a lipid across the knee, with the lipid class shown on the top right corner, and the m/z at which it was detected in the bottom left corner. Lipid species from left to right top to bottom were: PC (14:0/0:0); PC (16:1/0:0); PC (16:0/0:0); PC (18:1/0:0); PE(20:4/0:0); PC(18:1/0:0) – potassiated. AC = articular cartilage; C = AC and GP mix; CB = cortical bone; E = epiphyseal ROIs mix (marrow and subchondral bone); GP = growth plate; M = bone marrow.

The MALDI‐IMS pipeline was established using healthy knee joint tissue sections from juvenile C57BL/6 mice (Fig. 1*A*). MALDI‐IMS is a mass spectrometry–based imaging approach, where the tissue is divided into pixels and the mass spectrum of each pixel is established, resulting in a separate data point. The mass spectrum of each pixel consists of thousands of singly charged ions and their relative intensity. These ions are formed during laser desorption, which results in the attachment of adducts such as hydrogen (H), sodium (Na), and potassium (K) to the biomolecules present across the pixel. This process is assisted by the presence of a chemical matrix, which layers the tissue and can help with the selectivity of which compounds become ionized, in this case DHB matrix, which is common in lipidomic studies. Thus, the majority of ions in each mass spectrum are formed by the association of a lipid with H^+^ (protonated), Na^+^ (sodiated) or K^+^ (potassiated).

To assess data acquisition reproducibility and ion intensity changes within and across tissue types, analysis was carried out using sROIs and lROIs comprising 10 ablation pixels. To determine tissue specific ROIs, the MALDI‐IMS pixelated spectrum image was overlaid with a Safranin‐O/Fast Green FCF staining histological image, and ROIs were manually drawn based on histology. PCA was used for the unbiased exploration of the data and demonstrated a clear separation between the lipid profiles of bone, cartilage, and bone marrow ROIs in both sROI and lROI analyses (Fig. 1*B*). Thus, the more biased discriminant analysis modeling (OPLS‐DA) could be applied, because its assumptions that differences between the ROI groups exist on the tissue level were met. OPLS‐DA established the most significant contributor ions to the observed differential lipid profiles (Fig. 1*C*). Notably, the lROI analysis had a higher statistical power than the sROI analysis and highlighted the discriminant ions between tissues with a higher degree of confidence. Thus, lROI analysis was selected as the optimal approach for the untargeted comparison between tissues. Both sROI and lROI OPLS‐DA analyses reproducibly highlighted a group of 41 ions with differential distribution across tissues, of which 15 were strikingly prominent in the growth plate (Fig. S1 *A*). Subsequently, hierarchical clustering analysis was employed to understand the ability of these ions to differentiate ROIs on the tissue level. Similar results were observed across both the sROI and lROI analyses, with clearly defined clusters of cortical bone, bone marrow, and cartilage ROIs alongside ion clusters elevated in each of these tissue types (Fig. 1*D*). Interestingly, the articular cartilage and growth plate cartilage ROIs formed subclusters within the cartilage cluster (Fig. S1 *B*). In addition, within the growth plate cluster, some subclustering of hypertrophic zone ROIs was observed. This highlighted the potential of the pipeline in dissecting the lipid signatures of hypertrophic and articular cartilage.

An LC‐MS/MS–coupled approach was applied to assign lipid identification to the panel of 41 differentially abundant ions. This led to the identification of six ions prominent in the growth plate, including four LPCs and one lyso‐phosphatidylethanolamine (LPE) (Fig. 1*E*). LPC (16:0/0:0) was abundant across the entire joint but was most prominent in ligaments and the growth plate. LPC (14:0/0:0), LPC (16:1/0:0), LPC (18:1/0:0), and LPE (20:4/0:0) localized to the growth plate and articular cartilage, with the detected signal particularly high in the growth plate.

In summary, a MALDI‐IMS pipeline for data acquisition, ROI selection and subsequent statistical analysis was established. The tissue‐specific lipid signatures of cortical bone, bone marrow, growth plate, and articular cartilage were detected. Coupled with LC‐MS/MS, the pipeline identified a prominent lysophospholipid signature in the growth plate, which is an active site for mineralization in rapidly growing juvenile mice.

We hypothesized that these lysophospholipids play a role in the mineralization process, which slows down as mice mature. To test this hypothesis, we used the pipeline to map the lipidome of both adult and juvenile C57BL/6 mice.

### Spatial lipidomic profiles of the juvenile and adult mouse joint tissues

**Fig. 2:**
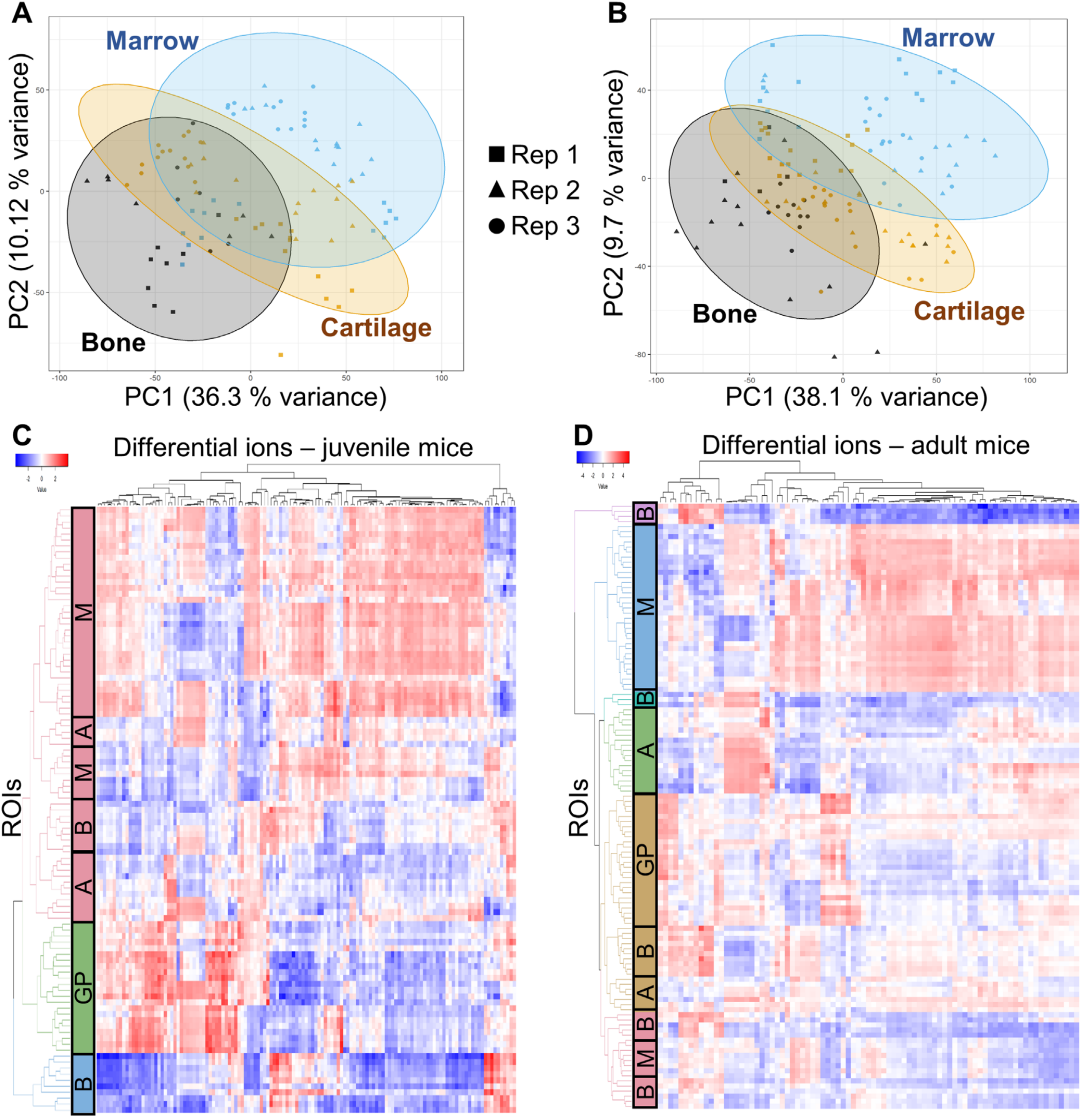
Mapping the lipidome of juvenile and adult mouse knee joints identifies differentially abundant ions across major tissues. PCA scores plots in (*A*) juvenile and (*B*) adult mice highlighted ROI grouping of bone, cartilage, and marrow ROIs, with subgrouping on the replicate level (different shapes represent different replicates). The ellipses indicate the Hotelling's T 95% confidence interval. Subsequent OPLS‐DA analysis generated a panel of 96 differentially abundant ions across growth plate, articular cartilage, cortical bone and bone marrow in juvenile mice (Fig. S2A–D) and a panel of 80 differential ions in adult mice (Fig. S3A–D). (*C*,*D*) Hierarchical clustering analysis demonstrated ROIs form tissue‐specific clusters based on the relative abundance of the ions in the generated panels. A = articular cartilage; B = cortical bone; GP = growth plate; M = marrow.

In both juvenile and adult mouse knees, the PCA highlighted ROI grouping on the tissue level, irrespective of the biological replicate (Fig. 2*A*,*B*). In juvenile mice, OPLS‐DA highlighted a panel of 96 differential ions across articular cartilage, growth plate cartilage, marrow, and cortical bone (Fig. S2*A*–*D*). Hierarchical clustering analysis demonstrated that these ions successfully segregate ROIs from the juvenile mouse joint in tissue‐specific clusters (Fig. 2*C*). The growth plate and bone marrow ROIs formed strikingly defined clusters across all replicates, and articular cartilage and cortical bone clustering was clearly observed within each replicate (Fig. S2*E*–*G*). In adult mice, the OPLS‐DA revealed a panel of 80 differential ions (Fig. S3*A*–*D*), which also successfully segregated adult joint ROIs into strong tissue‐specific clusters (Fig. 2*D*). Adult articular cartilage, growth plate cartilage, and bone marrow clusters were particularly prominent, and cortical bone clustering was observed within each replicate (Fig. S3*E*–*G*). Thus, our pipeline successfully defined the specific lipidomic profiles of cortical bone, bone marrow, and articular and growth plate cartilage in both adult and juvenile mice.

Of the 80 ions in the adult mouse panel, 69 overlapped with ions identified in the juvenile mouse data analysis. The two panels of differential ions were merged with the pipeline development panel, resulting in a final panel of 121 differential ions. Representative images of the distributions of these ions in adult and juvenile mice were assorted to generate a mouse knee joint lipid atlas (Fig. S4). The tissue‐specific lipid distribution signatures were similar in both age groups. As evident from the hierarchical clustering analysis, the majority of ions were enriched in either bone marrow or in growth plate cartilage, whereas cortical bone and articular cartilage exhibited a relatively lower abundance of lipids (Fig. 2*C*,*D*). Some of these ions were more prominent in tissues outside the joint, including muscles or ligaments, while a number of ions were elevated in the menisci and synovial membranes, such as m/z 782, m/z 784, and m/z 786.

### Establishing the mouse knee joint lipidomic signature by LC‐MS/MS–driven assignment

To explore the biomolecular differences across tissues of the joint, lipid identification was facilitated by our previously generated LC‐MS/MS dataset. Of all MALDI ions detected across our samples, 526 had putative hits in the LipidMatch database. These included lipid species spanning 17 lipid subclasses: acylcarnitines, ceramide phosphates, glucosylceramides, monogalactosyldiacylglycerols, phosphatidic acid, phosphatidylethanolamine (PE), PC, LPC, LPE, oxidized LPE, plasmanyl‐PC, plasmanyl‐LPC, plasmenyl‐PC, plasmenyl‐PE, plasmenyl‐LPC, sphingomyelin (SM), and triglycerides. All putative lipids with their relative intensity within tissues for juvenile and adult mice are available in Table S1. To our knowledge, this is the first spatial lipidomic fingerprinting of the mouse knee joint, with the lipid profile identified for articular cartilage, bone marrow, cortical bone, and growth plate cartilage. Of all putative hits, 44 lipids with differential abundance across tissues based on our multivariate analysis pipeline were manually annotated (Fig. S5). These were all lipids with conserved differential distribution in both adult and juvenile mice (Table S2). A further 35 differential ions had putative ID hits (Table S3), whereas the remaining 51 differential ions in the panel could not be identified.

**Fig. 3:**
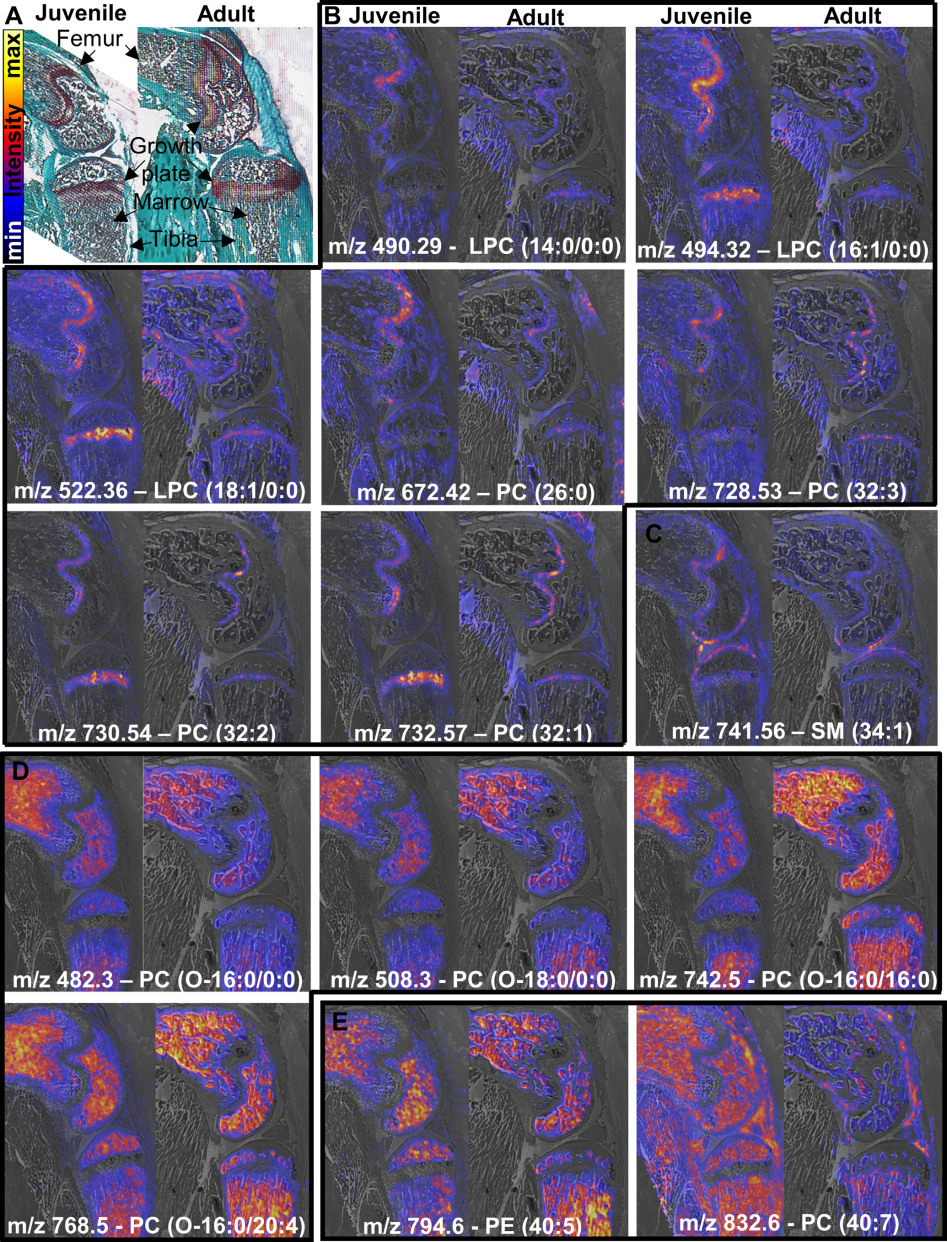
The growth plate and bone marrow lipid signature. Each image maps the distribution of a lipid across the juvenile and adult mouse knee, with the lipid notation and the m/z at which it was detected indicated at the bottom. (*A*) Schematic illustration of joint tissues and relative intensity scale. (*B*) Signature lipids in the growth plate were specific LPC and PC species. (*C*) SM (34:1) was strikingly prominent in articular cartilage. (*D*) Marrow was enriched in the platelet activating factors PC (O‐16:0/0:0) and PC (O‐18:0/0:0) and their related metabolites. (*E*) Signature lipids in marrow contained fatty acids with a high number of carbon atoms and double bonds. Representative images from adult and juvenile mice are shown side by side. *n* = 3 animals per group. Full panel of differential ions and distributions is available in Fig. S4.

Overall, growth plate cartilage and bone marrow had the most striking lipid profiles (Fig. 3*A‐E*). Phospholipids containing longer chain fatty acids (FAs) with a larger number of double bonds, PEs, and ether‐phospholipids were enriched in bone marrow (Fig. 3*D‐E*, Table S2). Four of the ether‐phospholipids in marrow were plasmanyl‐PCs comprising the PAFs LPC (O‐16:0/0:0) and LPC (O‐18:0/0:0) and their related metabolites PC (O‐16:0/16:0) and PC (O‐16:0_20:4). In comparison, the growth plate was enriched in specific PC and LPC species (Fig. 3*B*, Table S2). LPCs elevated in the growth plate contained the common FA chains palmitoleic acid (FA 16:1), myristic acid (FA 14:0), and oleic acid (FA 18:1), whereas LPCs enriched in marrow contained FA chains with a larger number of double bonds: linoleic acid (FA 18:2) and linolenic acid (FA 18:3) (Fig. 3*B‐E*, Table S2). Additionally, articular cartilage expressed a higher content of SM compared to the growth plate. SM (d34:1) was strikingly prominent in both adult and juvenile articular cartilage compared to the rest of the joint (Fig. 3*C*), whereas SM (d32:2) at m/z 813.6 and SM (d42:1) at m/z 815.7 were especially prominent in adult articular cartilage (Fig. S4).

Interestingly, the PCs enriched in the growth plate specifically had matched carbon and double bond counts that make them likely precursors of the observed elevated LPCs. For example, PC (32:2) cumulatively represents a number of PCs with 32 carbon atoms and two double bonds in their fatty acid chains, including PC (16:1/16:1), which can be converted to LPC (16:1/0:0) after cleavage of the sn‐2 acyl chain under phospholipase 2 (PLA2) activity.^(^ ^67^ ^)^ Furthermore, these PC and LPC species appeared more prominent in the juvenile mouse growth plates (Fig. S4), although this difference could not be quantified due to MALDI‐IMS–associated “batch effects” and lack of internal standard.^(^ ^68^ ^)^ This observation aligns with the hypothesis that the specific distribution of these PCs and LPCs in the growth plate is linked to biomineralization as proposed.^(^ ^51^, ^52^ ^)^ Therefore, we aimed to determine if PCs and LPCs enriched in the growth plate are involved in the PHOSPHO1‐mediated accumulation of P_i_.

### Spatial lipid profiling of the PHOSPHO1‐KO growth plate

**Fig. 4:**
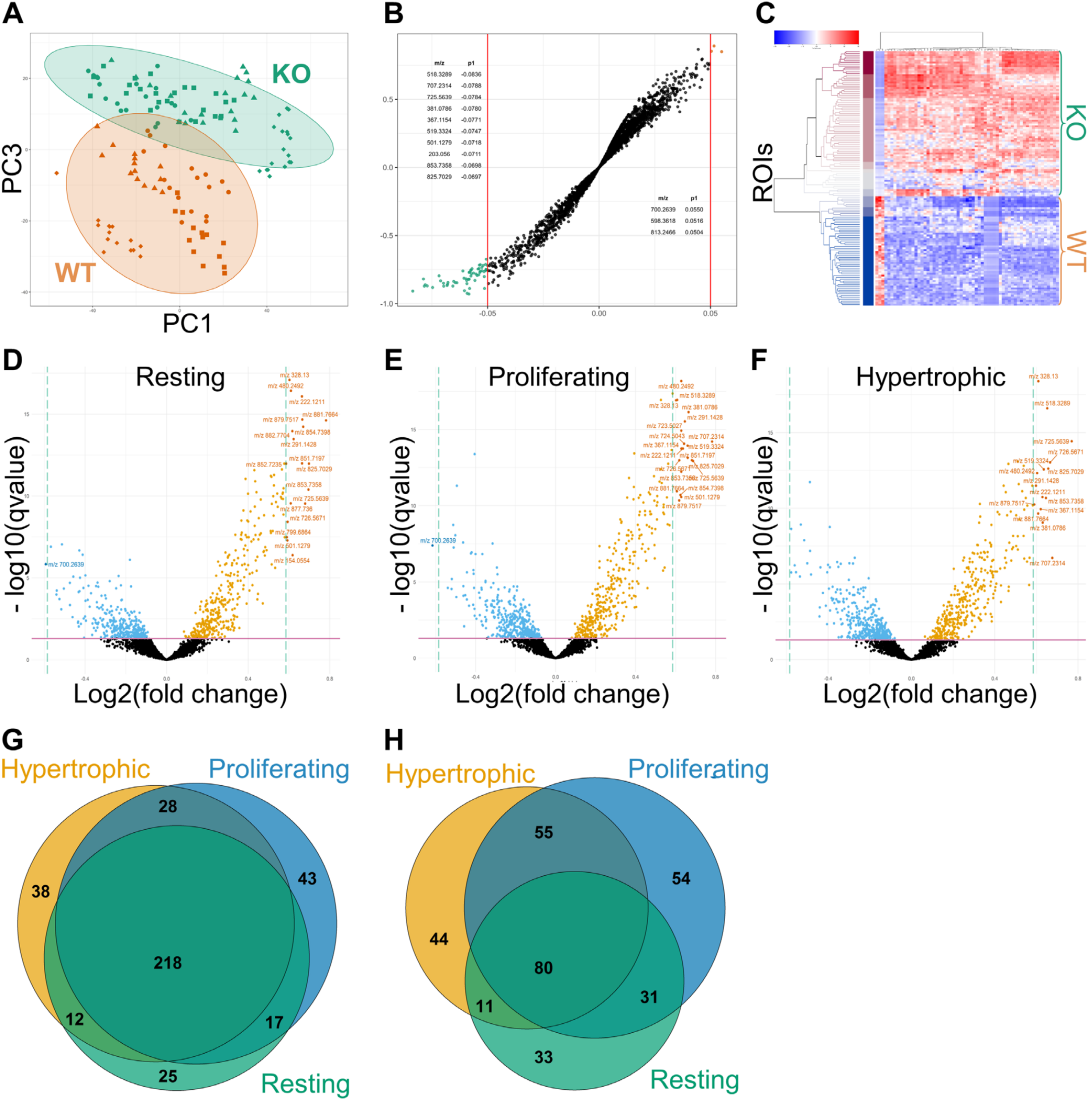
Significantly altered lipid ion signature of PHOSPHO1‐KO versus WT growth plates. (*A*) PCA scores plot of PC1 (22.18% variance) and PC3 (8.48% variance). Every point represents a growth plate ROI. Different shapes represent different biological replicates (*n* = 4). The ellipses indicate the Hotelling's T 95% confidence interval. (*B*) OPLS‐DA S‐plot of *p* value (*x*‐axis) against correlation (*y*‐axis). Every point represents an ion and the red lines represent a significance threshold of |p1| > 0.05. The highest contributor ions to differences between the WT (green) and KO (orange) growth plates are color‐coded. Tabulated are the top 10 ions elevated in each group, full list available in Table S4. (*C*) Hierarchical clustering analysis demonstrates segregation between WT and KO ROIs based on the top hits selected from the OPLS‐DA S‐plot. Each row represents and ROI, and each column represents a differential ion. Color‐coded is the abundance of each ion in standard deviations relative to the mean abundance of that ion across all ROIs. Blue represents values below the mean, white represents values at the mean, and red represents values above the mean. (*D*–*F*) Volcano plots of zone‐specific multiple *t* test analyses comparing WT control to PHOSHPO1‐KO growth plate zones. Each point represents an ion. The q‐value represents a false discovery rate (FDR)‐corrected *p* value. Ions upregulated in the KO have a log2(fold change) >0. The horizontal dotted line represents a 1.5‐fold‐change threshold. The significantly altered ions based on these analyses were used to generate Venn diagrams showing the overlap between (*G*) significantly upregulated and (*H*) significantly downregulated ions in the KO growth plate.

The MALDI‐IMS spatial lipidomics pipeline was applied to PHOSPHO1‐KO and WT control growth plate sections. For this experiment, SPLASH® LIPIDOMIX® internal standard, comprising deuterium‐labeled lipid species representative of a wide array of lipid classes was included as positive control and to enable normalization and relative quantification between the WT and KO mice. The growth plate was spatially resolved into resting, proliferating, and hypertrophic zones. PCA demonstrated a strong separation between all KO and WT growth plate ROIs based on principal component 3 (PC3), which accounted for 8.48% of the total variance (Fig. 4*A*). A similar separation was observed in the zone‐specific PCA scores plots (Fig. S6*A*–*C*). Subsequently, the OPLS‐DA analysis of the entire growth plate highlighted 660 differential ions (Table S4). The top hits with *p*(corr) > 0.5 based on the OPLS‐DA were established, resulting in a strong hierarchical clustering between WT and KO growth plate regions (Fig. 4*B*,*C*). Lipids were annotated against our LC‐MS/MS library of detected lipids, revealing an elevated presence of PCs, LPCs, and SM as top hits in the KO growth plate, except for LPC (22:2/0:0), which was enriched in the WT growth plate (Table S4).

Following this initial confirmation that ablation of PHOSPHO1 significantly alters the lipid landscape of the growth plate, analysis was further spatially resolved. The specific lipidomic signatures of resting, proliferating, and hypertrophic growth plate cartilage were interrogated across the WT and PHOSPHO1‐KO mice. Unpaired multiple *t* test was carried out for each growth plate zone, with the results displayed as volcano plots (Fig. 4*D*–*F*). A total of 381 ions were significantly upregulated in the PHOSPHO1‐KO, of which 218 were common to all zones (Fig. 4*G*), whereas 288 ions were significantly downregulated, of which 80 were common to all zones (Fig. 4*H*). The mean differences from the multiple *t* test analysis were used to calculate the fold change for each significant hit. Hits with fold change >1.5 were all upregulated in the PHOSPHO1‐KO. These included LPC (18:3/0:0), LPC (16:0/0:0), PC (38:2), and three SM species, which largely corresponded to the top hits across the entire growth plate, as previously established in the OPLS‐DA analysis (Table S4).

**Fig. 5:**
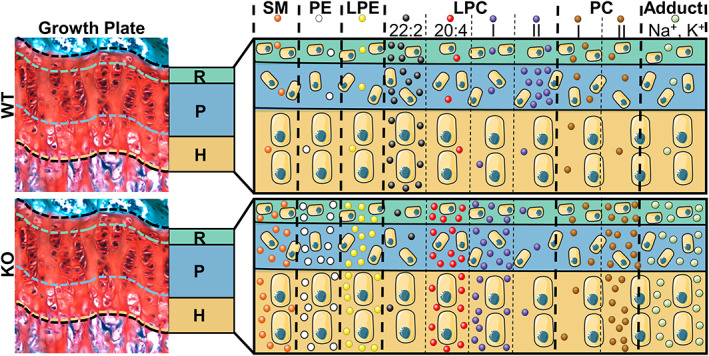
MALDI‐IMS of the PHOSPHO1‐KO growth plate reveals essential role of PHOSPHO‐1 in lipid homeostasis. Displayed is a schematic of the growth plate, with color‐coded resting (R), proliferating (P), and hypertrophic (H) zones. Each column represents a lipid class, with LPC and PC being split into sub‐groups based on patterns observed. The last column represents ion adducts. Upregulation and downregulation of lipids is represented by the relative abundance of the corresponding lipid circles. Some ions of some lipids exhibited zone‐specific distributions, as described in the Results section.

To gain a broader understanding of the lipid profile changes associated with PHOSPHO1 ablation, identification of all significant hits from the multiple *t* test analyses irrespective of fold change was carried out (Table S5). Overall, the PHOSPHO1‐KO growth plate was enriched in SM, PE, LPE, PC, LPC, sodiated ions, and potassiated ions, as schematically illustrated in Fig. 5.

SM, PE, and LPE species were elevated in the PHOSPHO1‐KO across all zones of the growth plate. Specifically, these included SM (d42:1), SM (d42:2), and SM (d48:1), PE (44:10), PE (38:5), PE (38:6), and PE (40:6), LPE (20:1/0:0), LPE (20:4/0:0), LPE (22:2/0:0), and LPE (22:6/0:0). LPCs could be split into two groups. The majority of LPCs were upregulated in the PHOSPHO1‐KO growth plate (group I), with LPC (20:4/0:0) the most confident assignment. An exception was LPC (22:2/0:0), which was downregulated across all zones of the KO growth plate. Group II of LPCs comprised LPCs with common fatty acid chains (16:0 – palmitic acid, 18:0 stearic acid, and 18:1 – oleic acid). Those LPCs were detected as enriched in the WT proliferating zone as protonated or dehydrated ions, whereas their sodiated ions were overall upregulated in the entire PHOSPHO1‐KO growth plate. Similar to LPCs, some protonated ions assigned as PCs were downregulated in the KO growth plate, but their sodiated and potassiated equivalents were upregulated in the KO compared to WT (PC group I). In contrast, group II of PCs comprised ions significantly upregulated exclusively in the KO growth plate versus WT, with specific distributions as follows: PC (34:1) was upregulated in the entire growth plate and especially in the resting zone, where three of its adduct ions were significantly enriched; PC (34:3) was upregulated in the proliferating zone; PC (36:4) was especially elevated in the resting zone but not the hypertrophic zone; PC (38:5) was especially prominent in the proliferating zone; PC (38:2), PC (40:7), PC (40:8), and PC (42:9) were upregulated in the entire KO growth plate. Other lipids significantly upregulated in the PHOSPHO1‐KO growth plate were glucosylceramide (d42:3) and acylcarnitine (18:1) (Table S5).

## Discussion

During mineralization, membrane lipids have been hypothesized to act as intermediates upstream of PHOSPHO1, a major contributor to the generation of P_i_ required for hydroxyapatite formation.^(^ ^68^ ^)^ However, crucial information about the precise spatial distribution of lipids in bone and joint tissues has been missing. This study aimed to spatially resolve the lipidome of the healthy mouse knee joint and to establish the effects of PHOSPHO1 ablation on growth plate lipid content and distribution.

A data acquisition and analysis pipeline was developed, combining MALDI‐IMS spatial profiling with LC‐MS/MS–driven lipidomics. This led to the creation of a lipid atlas for the knee joint, comprising differentially abundant lipid ions across the major joint tissues. Furthermore, 526 lipid ions were identified, including their relative abundance across each tissue in both juvenile and adult mouse knees, establishing a lipid biomolecular signature for articular and growth plate cartilage, cortical bone, and bone marrow. This revealed an enrichment of PAFs and related metabolites in bone marrow, LPC species in the growth plate, and SM in articular cartilage. The lipid fingerprint was conserved across both juvenile and adult mouse knees. We note that some variability may have been introduced in the analysis by grouping articular and growth plate cartilage during the cartilage versus marrow and cartilage versus cortical bone OPLS‐DA (Fig. S2*B*,*C*). This limitation was remediated by exploring the differences between growth plate and articular cartilage (Fig. S2*D*). When applied to PHOSPHO1‐KO mouse knees, the pipeline demonstrated a significant alteration in the lipid profile of the growth plate across resting, proliferating and hypertrophic zones. Specifically, this means an enrichment of SM, PE, LPE, PC, LPC, sodiated ions, and potassiated ions was observed in the PHOSPHO1‐KO, suggesting a role of PHOSPHO1 in lipid homeostasis. However, it is important to note that MALDI‐IMS is a semiquantitative technique. Therefore, further functional validation and quantification via independent approaches is needed to solidify these results.

The spatially‐resolved lipid profile of the joint presented here is in line with tissue‐specific functions. The high presence of PAFs and related metabolites in marrow could be associated with the importance of PAF signaling in adipose tissue remodeling^(^ ^69^ ^)^ and as mediators of inflammation.^(^ ^70^ ^)^ Similarly, the prevalence of phospholipids containing long‐chain polyunsaturated fatty acids in marrow can be associated with the energy‐storage function of adipocytes. Linoleic acid and linolenic acid are the two most abundant polyunsaturated fatty acids. They act as precursors of the longer‐chain polyunsaturated fatty acids dihomo‐γ‐linolenic acid, arachidonic acid, eicosapentaenoic acid, and docosahexaenoic acid, making them essential mediators of energy expenditure.^(^ ^71^ ^)^ In this study we demonstrated that lysophospholipids containing linoleic and linolenic acid were especially prominent in marrow, which reflects the importance of phospholipid membrane components not only in structural functions but also in metabolic and regulatory signaling.

Importantly, free fatty acids (FFAs) released from adipocytes act as local signaling mediators of osteoclasts and osteoblasts.^(^ ^72^ ^)^ Notably, the FFAs palmitate and palmitoleate have been implicated in Wnt signaling through their involvement in posttranslational modifications of Wnt ligands.^(^ ^6^ ^)^ Palmitoylation of a Wnt protein cysteine residue is required to enable binding to Frizzled receptors and activation of downstream signaling,^(^ ^72^, ^73^ ^)^ and both canonical and noncanonical Wnt signaling pathways have a major influence on bone cell behavior and cartilage development.^(^ ^73^, ^74^ ^)^ It is possible that in addition to fulfilling a signaling role in the Wnt pathways, the FFAs released by adipocytes are then re‐cycled to generate membrane phospholipids in the growth plate. This could explain why PCs and LPCs containing the FA 16:0 (palmitate) and FA 16:1 (palmitoleate) were especially prominent in the growth plate, where chondrocytes play a crucial role in the ossification process, which is central to bone maintenance.

Despite the importance of hypertrophic ossification in normal bone development and growth, ectopic ossification of articular cartilage is a common undesirable phenotype associated with osteochondral pathologies,^(^ ^75^ ^)^ the most prevalent being OA. Given the importance of lipids in regulating the ossification process, it is unsurprising that OA has been linked to altered FFA content in all tissues of the joint, including cartilage, synovium and infrapatellar fat pad.^(^ ^76^, ^77^, ^78^ ^)^ In OA, articular chondrocytes are enlarged and have an increased apoptotic rate, similar to hypertrophic chondrocytes. This phenotype has been linked to an excessive number of terminally differentiated chondrocytes in OA compared to healthy cartilage, where terminal chondrocyte differentiation is suppressed. In this study we established the lipid signature of both healthy hypertrophic (growth plate) and articular cartilage. Our findings provide a number of SM biomarkers elevated in articular cartilage compared to hypertrophic cartilage. This implies a biologically relevant involvement of SM in the manifestation of an articular versus hypertrophic chondrocyte phenotype. One explanation for the increased levels of SM in articular chondrocytes could be the desirable properties of SM as a boundary lubricant, contributing to the lubricity and durability of articular cartilage during joint movement.^(^ ^79^, ^80^ ^)^ Another explanation could be the involvement of SM catabolism in ECM synthesis and biomineralization. SM is catabolized by sphingomyelinases (SMases) to generate PCho and ceramide. In articular chondrocytes, SM catabolism has been shown to contribute to ECM remodeling and homeostasis.^(^ ^81^ ^)^ More specifically, treatment with exogenous SMase resulted in a dose‐dependent significant increase in proteoglycan release, but also significantly increased ceramide‐induced apoptosis. As previously reviewed, in hypertrophic chondrocytes hydrolysis of SM by neutral sphingomyelinase 2 (nSMase2) generates endogenous ceramide necessary for the normal apoptosis associated with terminal chondrocyte differentiation.^(^ ^82^ ^)^ Thus, the differential abundance of SM in articular versus hypertrophic cartilage could be the result of a combination of two factors. First, SM may be preferentially generated and incorporated in the cell membranes of articular chondrocytes via an unknown mechanism to improve lubricity. Second, SM catabolism is likely upregulated in hypertrophic chondrocytes, where the products of this process are required for apoptosis associated with the terminal hypertrophic stage.

In addition to its involvement in ceramide‐induced apoptosis, SM catabolism is central to physiological mineralization. This has been demonstrated in mice by deletion of the gene encoding nSMase2, resulting in a severely hypomineralized cortical bone.^(^ ^83^ ^)^ It has been proposed that the PCho generated as product of SM hydrolysis by nSMase2 is subsequently hydrolyzed by PHOSPHO1 to liberate P_i_ within MVs required for sufficient mineralization.^(^ ^84^ ^)^ In addition to the SM‐nSMase2 pathway, two other pathways involving membrane lipids upstream of the PHOSPHO1‐mediated accumulation of P_i_ have been proposed, as recently reviewed.^(^ ^85^ ^)^ These pathways are analogous, whereby PC or PE membrane lipids are hydrolyzed to LPC or LPE, respectively, by PLA2, and the LPC and LPE products are then further hydrolyzed by ENPP6 to generate the PHOSPHO1 substrates PCho and PEtOH, respectively.^(^ ^85^ ^)^

**Fig. 6:**
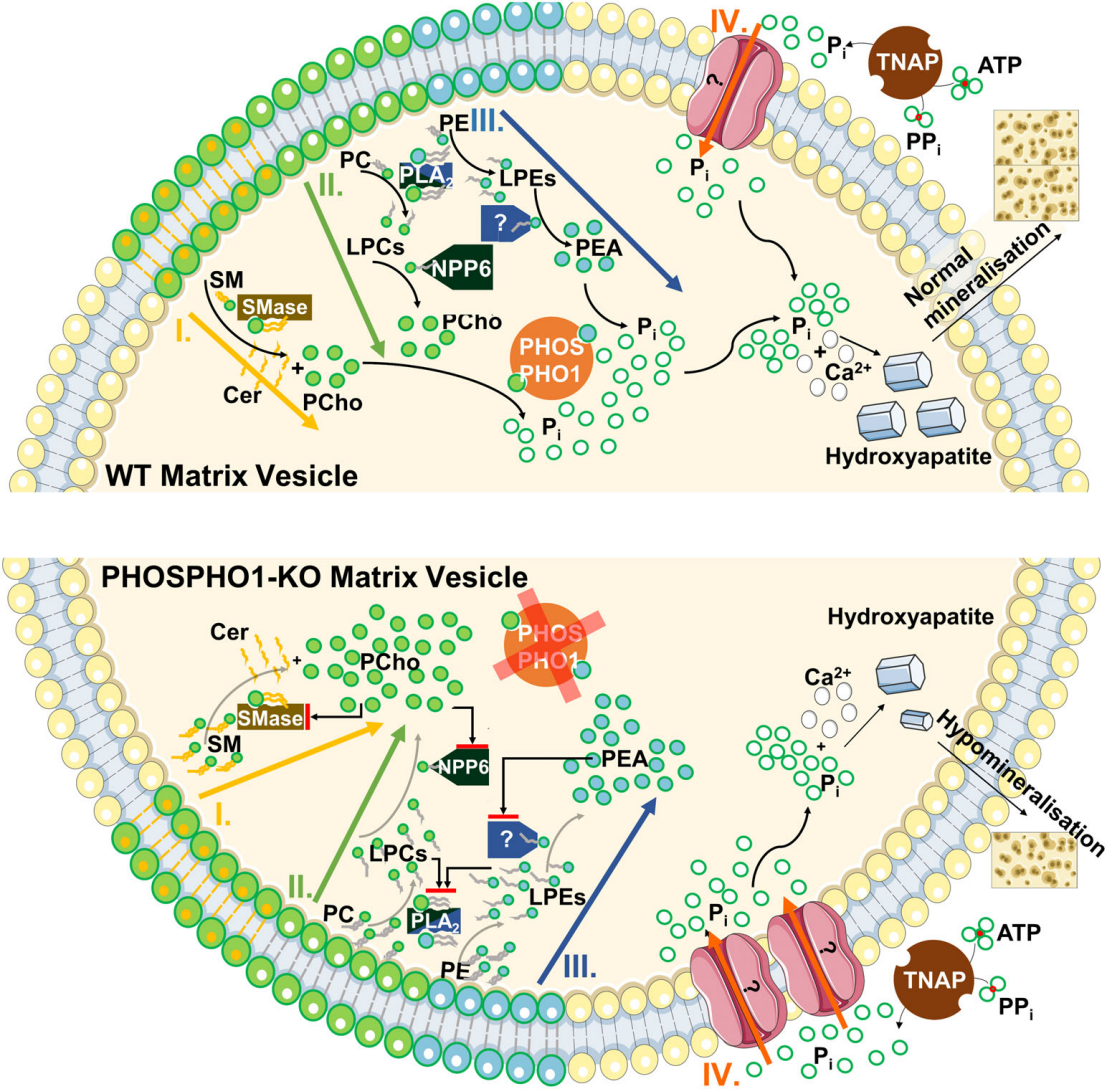
Hypothesized PHOSPHO1 biochemical pathways in MVs from WT (top) and PHOSPHO1‐KO (bottom) mice. Pathways are schematically illustrated. Part I: In WT mice SM from the MV membrane is hydrolyzed to PCho under the action of Smase2, resulting in a ceramide by‐product. In PHOSPHO1‐KO mice, accumulation of unmetabolized PCho inhibits Smase2 and leads to an accumulation of unhydrolyzed SM. Part II: In WT mice, PLA2 hydrolyses PC to LPC, which is then converted to PCho under the action of NPP6, resulting in the net release of 2 FFA. In PHOSPHO1‐KO mice, accumulation of unmetabolized PCho inhibits NPP6 and leads to an accumulation of LPCs, which in turn inhibits further hydrolysis of PCs by PLA2, leading to an increased PC content. Part III: In the WT mouse PE is converted to LPE by PLA2, then phosphoethanolamine (PEA) is liberated from the generated LPE under the action of an unknown enzyme analogous to NPP6. When PHOSPHO1 is ablated, accumulation of PEA inhibits the unknown enzyme activity, leading to an accumulation of LPE, in turn inhibiting PLA2 hydrolysis of PE and leading to an increased PE content. Part IV: In the WT mouse, extracellular P_i_ is also generated by TNAP from ATP and pyrophosphate (PP_i_). It is known that P_i_ moves into the lumen from extracellular sources,^(^ ^87^, ^88^ ^)^ but the mechanisms remain unclear. This, together with the actions of PHOSPHO1, helps achieve the high P_i_ concentrations required for the formation of hydroxyapatite crystals, which are then released into the ECM to generate the mineralized bone matrix. In PHOSPHO1‐KO mice, these mechanisms may be upregulated in an attempt to rescue the resulting phenotype via increased influx of TNAP‐generated P_i_, resulting in some, but insufficient hydroxyapatite and hypomineralization.

To dissect these biochemical pathways further, we determined the differences in lipid content in the growth plates of WT and PHOSPHO1‐KO mice. For the first time, our data provide evidence to support the existence of all three proposed pathways contributing to the simultaneous generation of P_i_ within MVs, as described in the previous paragraph. More specifically, our data suggest P_i_ generation and mineralization in the growth plate is highly dependent on the biochemical digestion of the membrane lipids SM, PC and PE, and on the products resulting from this enzymatic digestion—LPE and LPC. This is demonstrated by the zone‐dependent increase in SM, PE, PC, LPE, and LPC in the PHOSPHO1‐KO growth plate compared to WT (Fig. 5). These findings are further supported by early studies of the lipid composition of the membrane of MVs found in cartilage. Wu and colleagues^(^ ^85^ ^)^ have demonstrated that the MV membrane is rich in PC and PE species, which progressively decline over time during mineralization. We propose that the accumulation of phospholipids we observed in the PHOSPHO1‐KO growth plate is the result of a two‐step negative feedback loop that limits the digestion of the membrane phospholipids PC, PE, and SM (Fig. 6). First, lack of PHOSPHO1 in the growth plate results in accumulation of its substrates, PEtOH and PCho. This accumulation triggers a negative feedback loop, preventing further hydrolysis of SM, LPC, and LPE. In turn, this leads to an accumulation of LPCs and LPEs, which triggers another negative feedback loop, preventing further hydrolysis of PC and PE by PLA2. As a result, an accumulation of SM, PC, PE, LPE, and LPC remain undigested in the PHOSPHO1‐KO growth plate, leading to the significantly higher levels at which we detect these lipid classes in the KO compared to WT mice. Demonstrating the existence of such negative feedback loops through functional studies and further examining how the observed lipidome changes cause hypomineralization will be important next steps in elucidating the role of PHOSPHO1 and lipid metabolism in bone mineralization.

We also demonstrated that for some PCs and LPCs, sodiated and potassiated ions were preferentially formed in the PHOSPHO1‐KO mice, as opposed to protonated and dehydrated ions in the WT mice. It has been postulated that Pi transport from the ECM to the MV lumen may be performed by a type III Na/Pi transporter co‐transporter (PiT‐1/Glvr1) encoded by the *Slc20a1* gene.^(^ ^44^, ^86^, ^87^ ^)^ The presence of PiT‐1 in isolated chick MVs was suggested on the basis of Pi transport activity.^(^ ^86^, ^87^ ^)^ Furthermore, we have previously reported that mice deficient in both PHOSPHO1 and PiT‐1 have a hypomineralized skeleton that is worse than that noted in PHOSPHO1 KO mice.^(^ ^87^, ^88^, ^89^ ^)^ However, several proteomic studies have been unable to detect PiT‐1, PiT‐2, nor any other sodium‐coupled phosphate transporter within MV.^(^ ^88^, ^89^, ^90^ ^)^ Furthermore, no sodium gradient exists and there are no studies reporting the expression of a functional Na/K pump within MVs.^(^ ^91^ ^)^ Therefore, it is possible that the accumulation of sodiated ion adducts within PHOSPHO1‐KO growth plate may be a consequence of an alteration in ion‐dependent transporters such as Na+/K+ ATPase α1; SLC12A2 (Na+/K+/Cl‐ transporter); SLC4A7 (sodium bicarbonate cotransporter), which have been reported to be present in MVs by proteomic studies.^(^ ^88^, ^89^, ^90^ ^)^

Our imaging pipeline has established a spatially‐resolved lipid profile of joint tissues and generated an atlas of tissue‐specific biomarkers which may be used, for example, to track tissue integrity and disease status. We have demonstrated the role that PHOSPHO1 plays in the growth plate lipidome, highlighting its essential function in lipid homeostasis and bone health.

## Author Contributions

JT, PG, SD, CF, TL, AJR, and CDB contributed to conceptualization. The methodology, investigations, visualization and validation were carried out by JT under the supervision of PG, TL, CF, AJR, and CDB. Data was curated and formally analyzed by JT and TL. Resources were provided by PG, TL, JLM, LAS and CF. TL and JT contributed to software used in this study. The original draft was prepared by JT and reviewed and edited by all authors. PG was responsible for project administration.

## Disclosures

PG is co‐founder and Chief Scientific Officer for the University of York spin‐out company Mesenbio. The remaining authors have no conflicts of interest.

### Peer Review

The peer review history for this article is available at https://publons.com/publon/10.1002/jbmr.4796.

## Supporting information


**Fig. S1.** Supplementary figures
Figs. S1–S6

**Table S1.** The lipid signature of bone marrow, articular catilage, cortical bone, and the growth plate in juvenile and adult mice.

**Table S2.** Manually annotated differential lipids of the mouse knee.

**Table S3.** Differential lipids of the mouse knee with putative ID.

**Table S4.** Ions with differential abundance in the WT vs PHOSPHO1‐KO growth plate.

**Table S5.** Spatially resolved zone‐specific lipidomic changes in the PHOSPHO1‐KO vs WT growth plate.

## Data Availability

The data that supports the findings of this study are available in the supplementary material of this article. The raw lipidomics data and supplementary material have been deposited to the ProteomeXchange Consortium via the PRIDE^(^ ^92^ ^)^ partner repository with the dataset identifier PXD038909.
