# Supplementary material for: Spatial Lipidomic Profiling of Mouse Joint Tissue Demonstrates the Essential Role of PHOSPHO1 in Growth Plate Homeostasis
Source: J Bone Miner Res. 2023 Mar 14;38(5):792–807. doi: 10.1002/jbmr.4796 (PMC10946796; doi:10.1002/jbmr.4796)
Supplement: Supplementary file 1 — Fig. S1. Supplementary figures Figs. S1–S6 [file JBMR-38-792-s005.pdf]

Fig. S1A

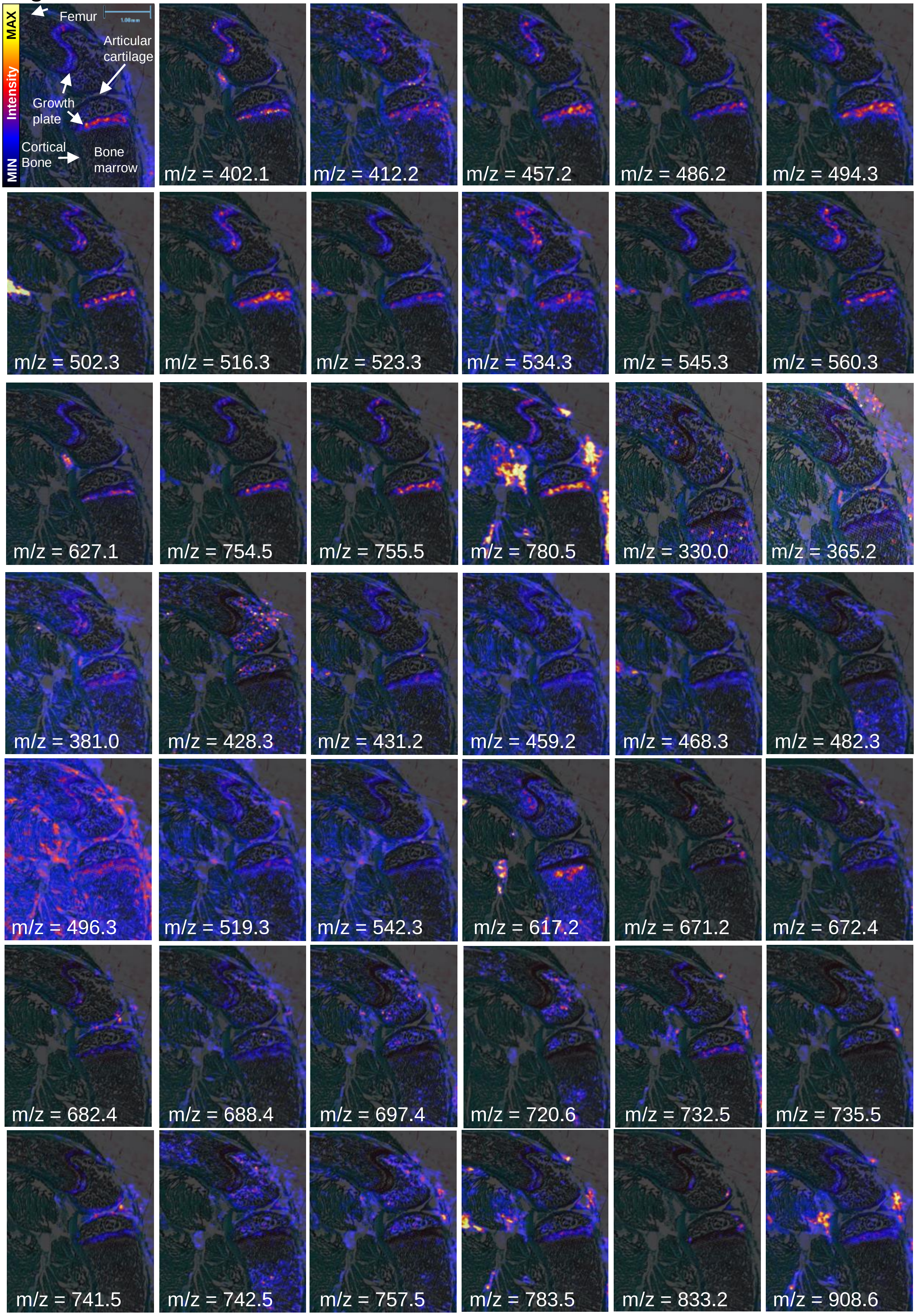

Differentially abundant ions across major knee joint tissues**.** Raw ion intensity distribution is colour-coded relative to the minimum (cold colours) and maximum (hot colours) for that ion. Ions strikingly prominent in the growth plate are displayed in the top 3 rows.

Fig. S1B

Enlarged hierarchical clustering analysis heatmaps with added colour-coded annotation for ROIs on the tissue and sub-tissue level, as indicated in the legend below. The heatmaps include ROIs from all biological replicates (n=3), and the heat colour code represents z-score normalized ion intensity.

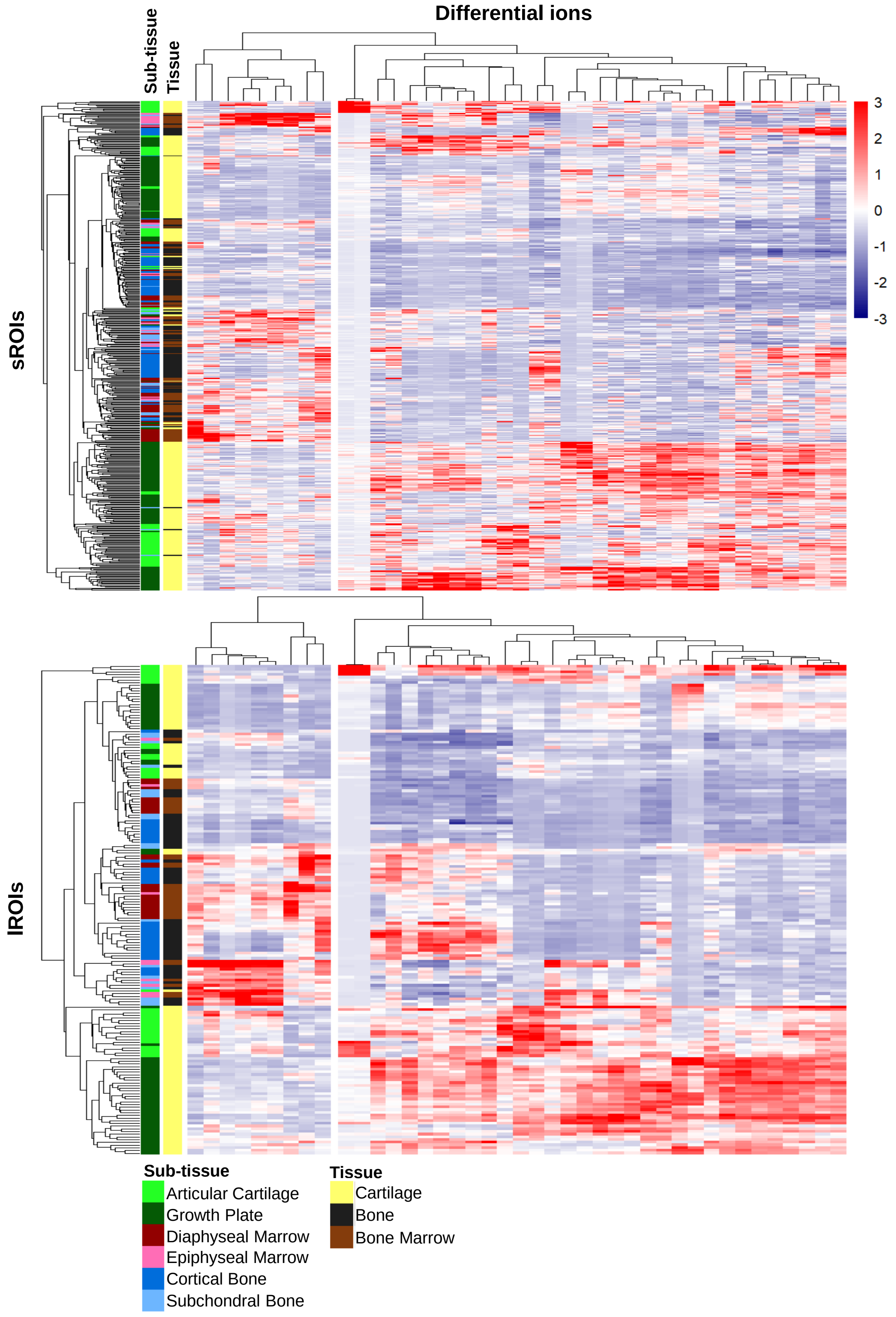

Fig. S2

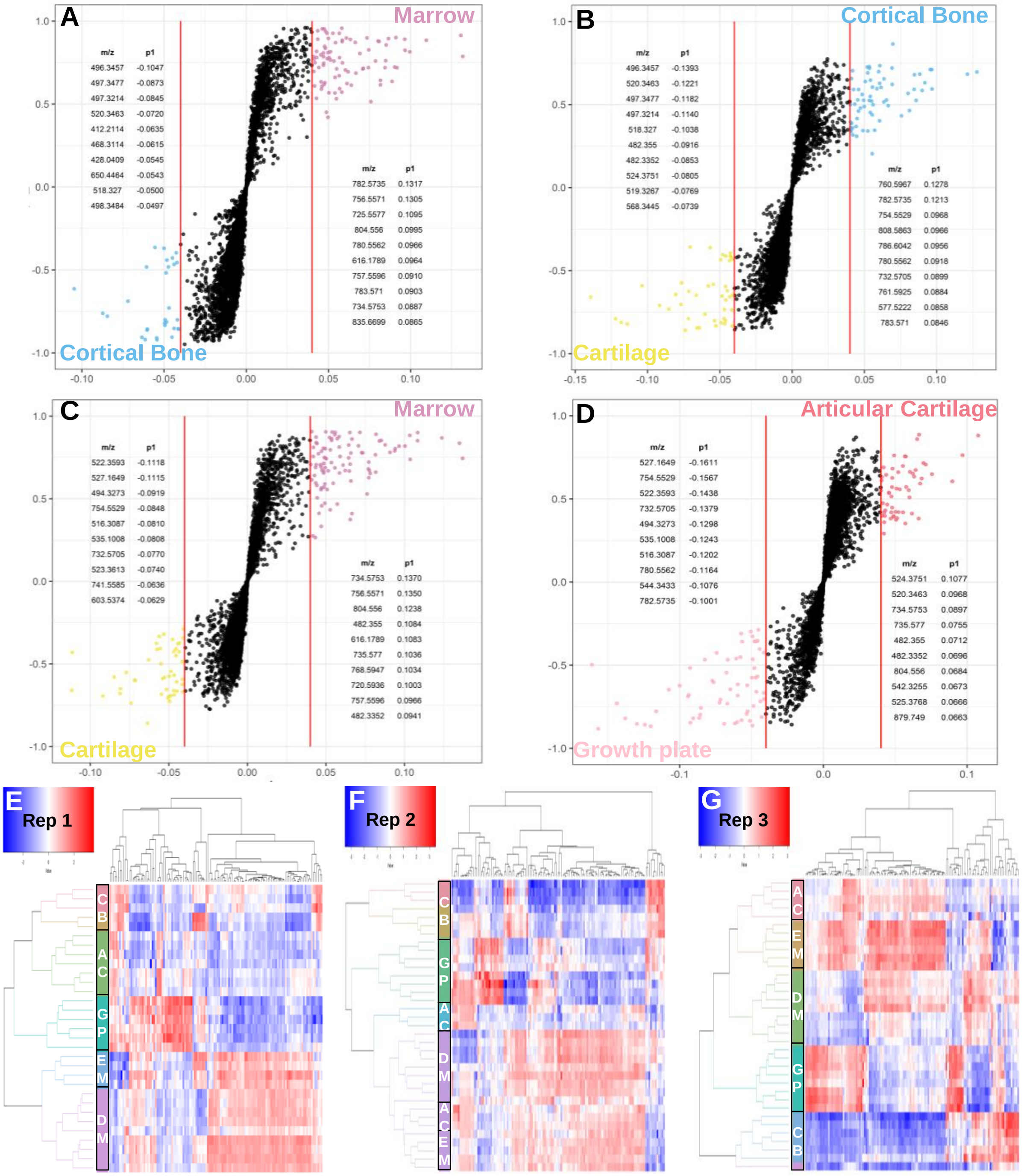

Generating the juvenile mouse knee lipidome panel of differential ions; replicate-dependent tissue-specific segregation of ROIs. OPLS-DA analysis generated a panel of 96 differentially abundant ions across growth plate, articular cartilage, cortical bone and bone marrow in juvenile mice. **A-D** The resulting S-plots of covariance (x-axis) plotted against correlation (y-axis) are shown. Each dot represents an ion, and the red lines represent a significance threshold of |p1|>0.04. The top 10 discriminant ions elevated in each tissue are tabulated. ROIs were analysed in tissue pairs: **a** cortical bone versus marrow, **B** cartilage versus cortical bone, **C** cartilage versus bone marrow, **D** articular cartilage versus growth plate cartilage. **E-G** Subsequent hierarchical clustering based on the generated panel of differential ions demonstrated strong tissue-specific segregation within each replicate. The heatmap colour coding represents z-score normalised difference from the mean, with blue showing relative abundance of ions below the mean and red showing high relative abundance. Abbreviations: AC – Articular Cartilage; GP – Growth Plate; CB – Cortical Bone; M – Marrow; D – Diaphyseal; E – Epiphyseal

Fig. S3

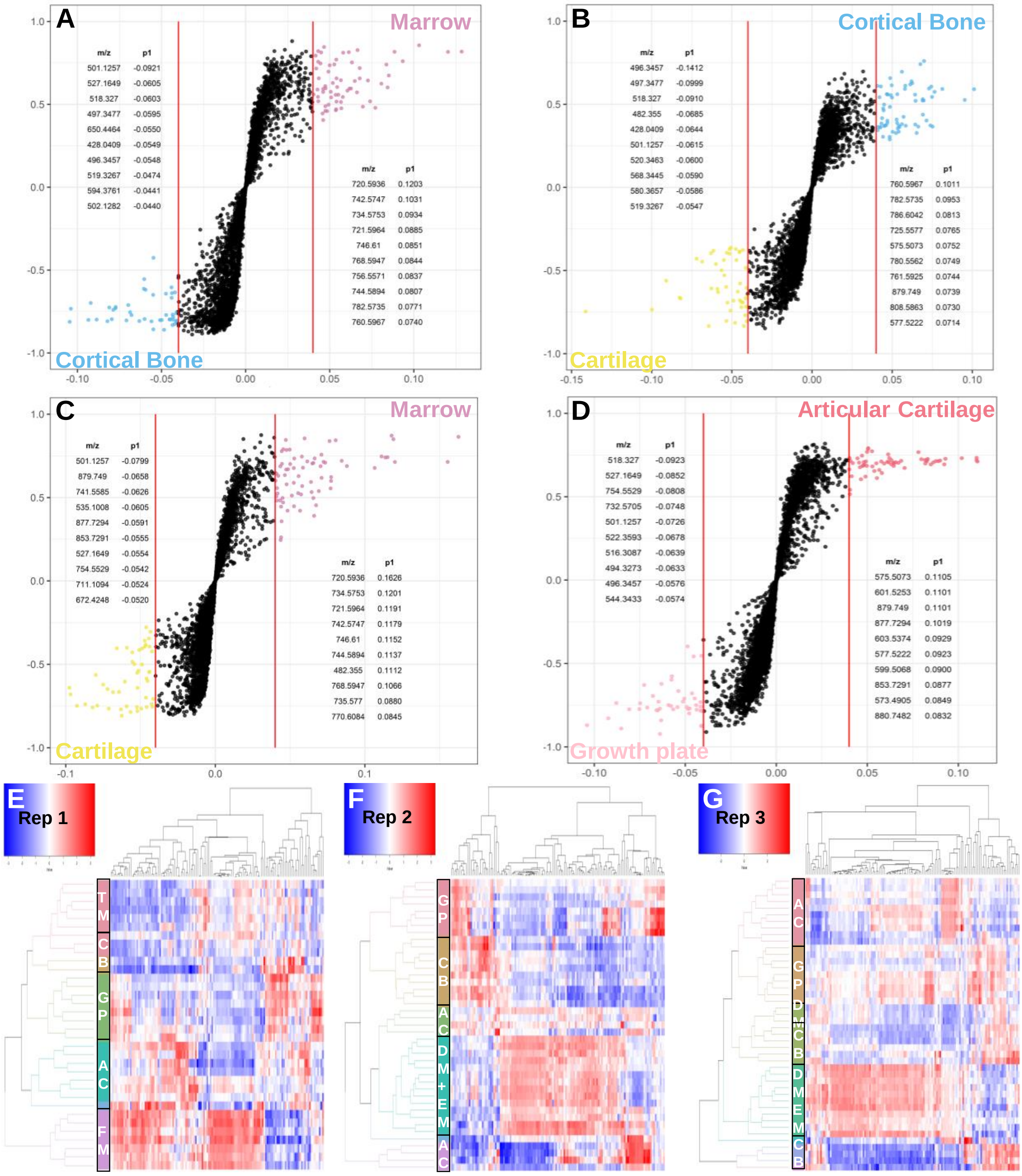

Generating the adult mouse knee lipidome panel of differential ions; replicate-dependent tissue-specific segregation of ROIs. OPLS-DA analysis generated a panel of 80 differentially abundant ions across growth plate, articular cartilage, cortical bone and bone marrow in adult mice. **A-D** The resulting S-plots of covariance (x-axis) plotted against correlation (y-axis) are shown. Each dot represents an ion, and the red lines represent a significance threshold of |p1|>0.04. The top 10 discriminant ions elevated in each tissue are tabulated. ROIs were analysed in tissue pairs: **a** cortical bone versus marrow, **B** cartilage versus cortical bone, **C** cartilage versus bone marrow, **D** articular cartilage versus growth plate cartilage. **E-G** Subsequent hierarchical clustering based on the generated panel of differential ions demonstrated strong tissue-specific segregation within each replicate. The heatmap colour coding represents z-score normalised difference from the mean, with blue showing relative abundance of ions below the mean and red showing high relative abundance. Abbreviations: AC – Articular Cartilage; GP – Growth Plate; CB – Cortical Bone; M – Marrow; D – Diaphyseal; E – Epiphyseal; T – Tibial; F – Femoral

Fig. S4

The mouse knee lipid atlas of differential ions. Includes representative images from adult and juvenile mouse knee cross sections for all 121 ions with differential abundance across bone marrow, cortical bone, articular cartilage, and growth plate cartilage. Where available, manually annotated or putatively annotated lipid ID is recorded, for more information refer to Table S2 and Table S3. Where lipid ID is separated by | there are multiple possible ID hits for that ion. Where no lipid ID is stated, the ion could not be identified.

Abbreviations and nomenclature:

ND	–	not detected
PC	–	Phosphatidylcholine
O-PC	–	Plasmanyl-PC
LPC	–	Lyso-phosphatidylcholine
O-LPC	–	Plasmanyl-Lyso-phosphatidylcholine
PE	–	Phosphatidylethanolamine
LPE	–	Lyso-phosphatidylethanolamine
SM	–	Sphingomyelin
FA	–	Fatty acid

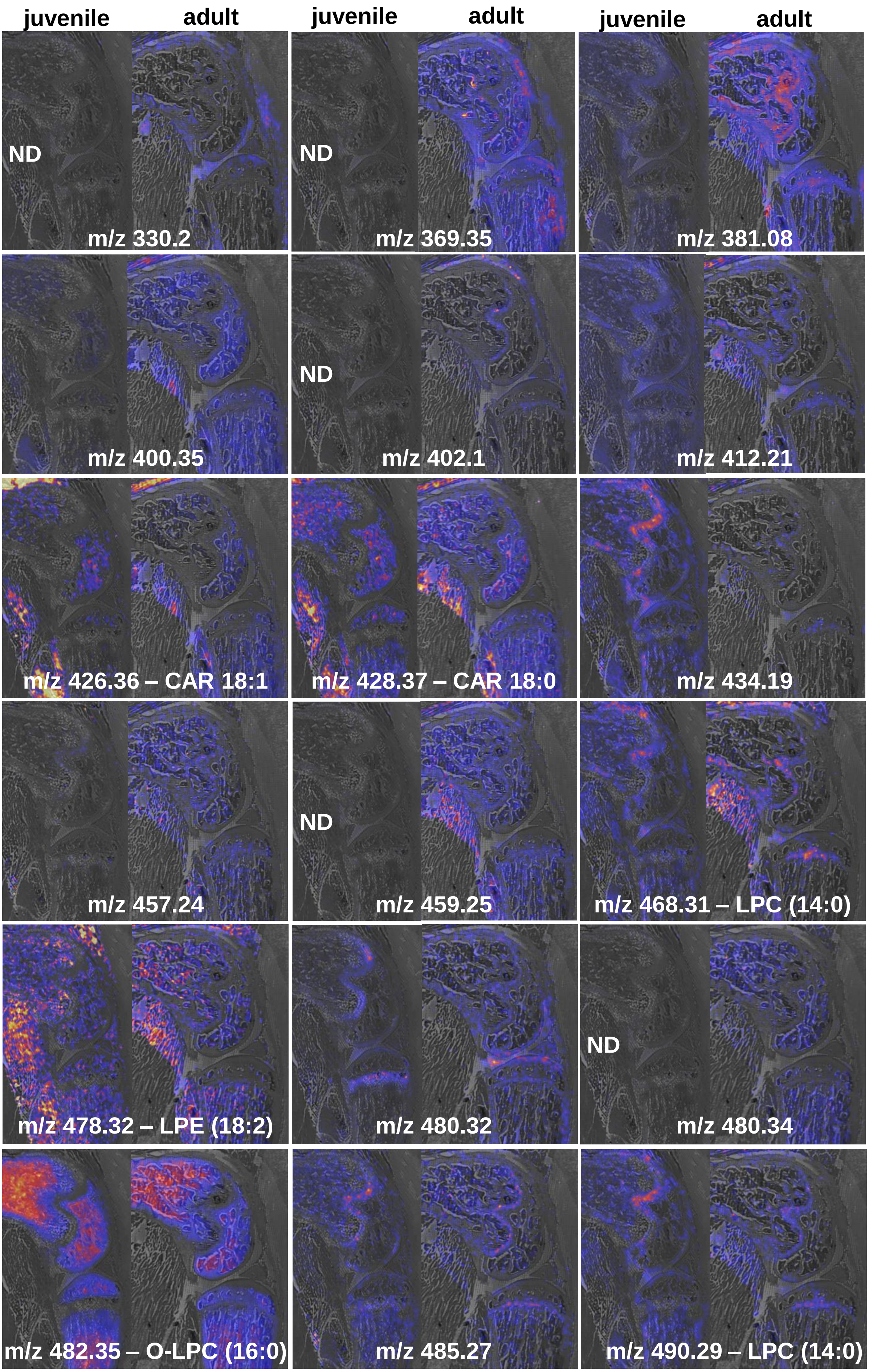
juvenile
adult
juvenile
adult
juvenile
adult
ND
m/z 330.2
ND
m/z 369.35
m/z 381.08
m/z 400.35
ND
m/z 402.1
m/z 412.21
m/z 426.36 – CAR 18:1
m/z 428.37 – CAR 18:0
m/z 434.19
m/z 457.24
ND
m/z 459.25
m/z 468.31 – LPC (14:0)
m/z 478.32 – LPE (18:2)
m/z 480.32
ND
m/z 480.34
m/z 482.35 – O-LPC (16:0)
m/z 485.27
m/z 490.29 – LPC (14:0)

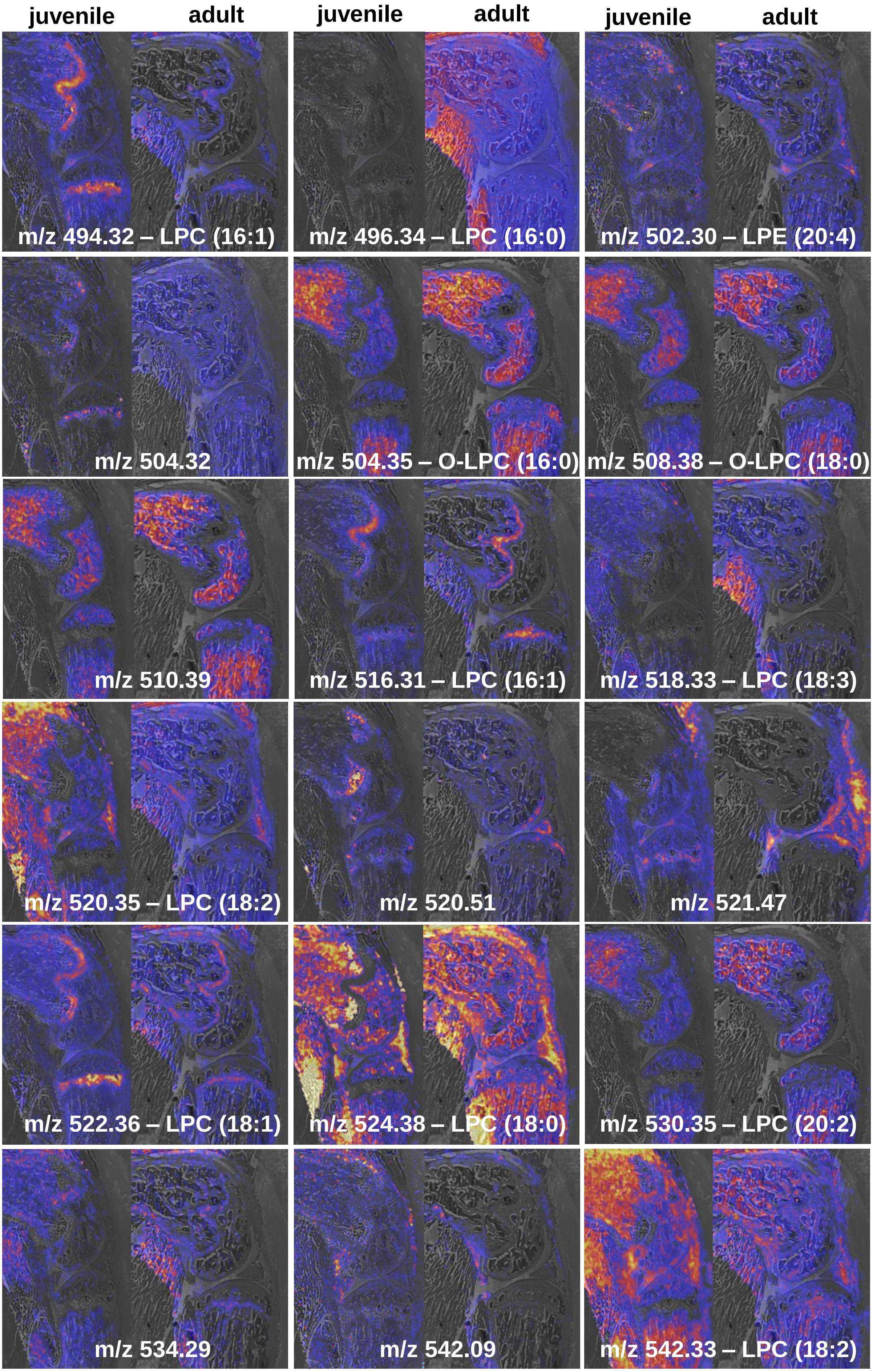
juvenile
adult
juvenile
adult
juvenile
adult
m/z 494.32 – LPC (16:1)
m/z 496.34 – LPC (16:0)
m/z 502.30 – LPE (20:4)
m/z 504.32
m/z 504.35 – O-LPC (16:0)
m/z 508.38 – O-LPC (18:0)
m/z 510.39
m/z 516.31 – LPC (16:1)
m/z 518.33 – LPC (18:3)
m/z 520.35 – LPC (18:2)
m/z 520.51
m/z 521.47
m/z 522.36 – LPC (18:1)
m/z 524.38 – LPC (18:0)
m/z 530.35 – LPC (20:2)
m/z 534.29
m/z 542.09
m/z 542.33 – LPC (18:2)

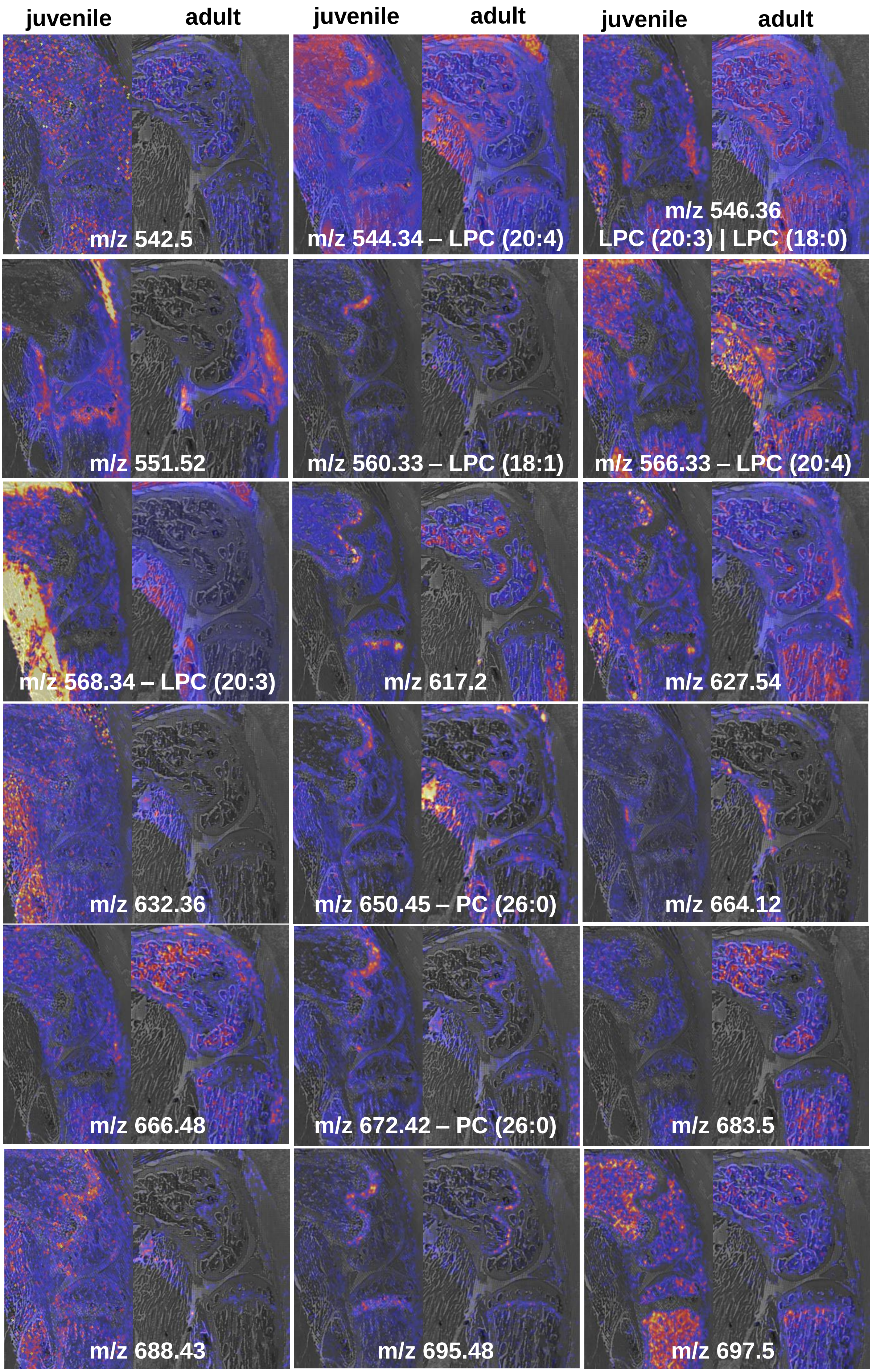
juvenile
adult
juvenile
adult
juvenile
adult
m/z 542.5
m/z 544.34 – LPC (20:4)
m/z 546.36
LPC (20:3) | LPC (18:0)
m/z 551.52
m/z 560.33 – LPC (18:1)
m/z 566.33 – LPC (20:4)
m/z 568.34 – LPC (20:3)
m/z 617.2
m/z 627.54
m/z 632.36
m/z 650.45 – PC (26:0)
m/z 664.12
m/z 666.48
m/z 672.42 – PC (26:0)
m/z 683.5
m/z 688.43
m/z 695.48
m/z 697.5

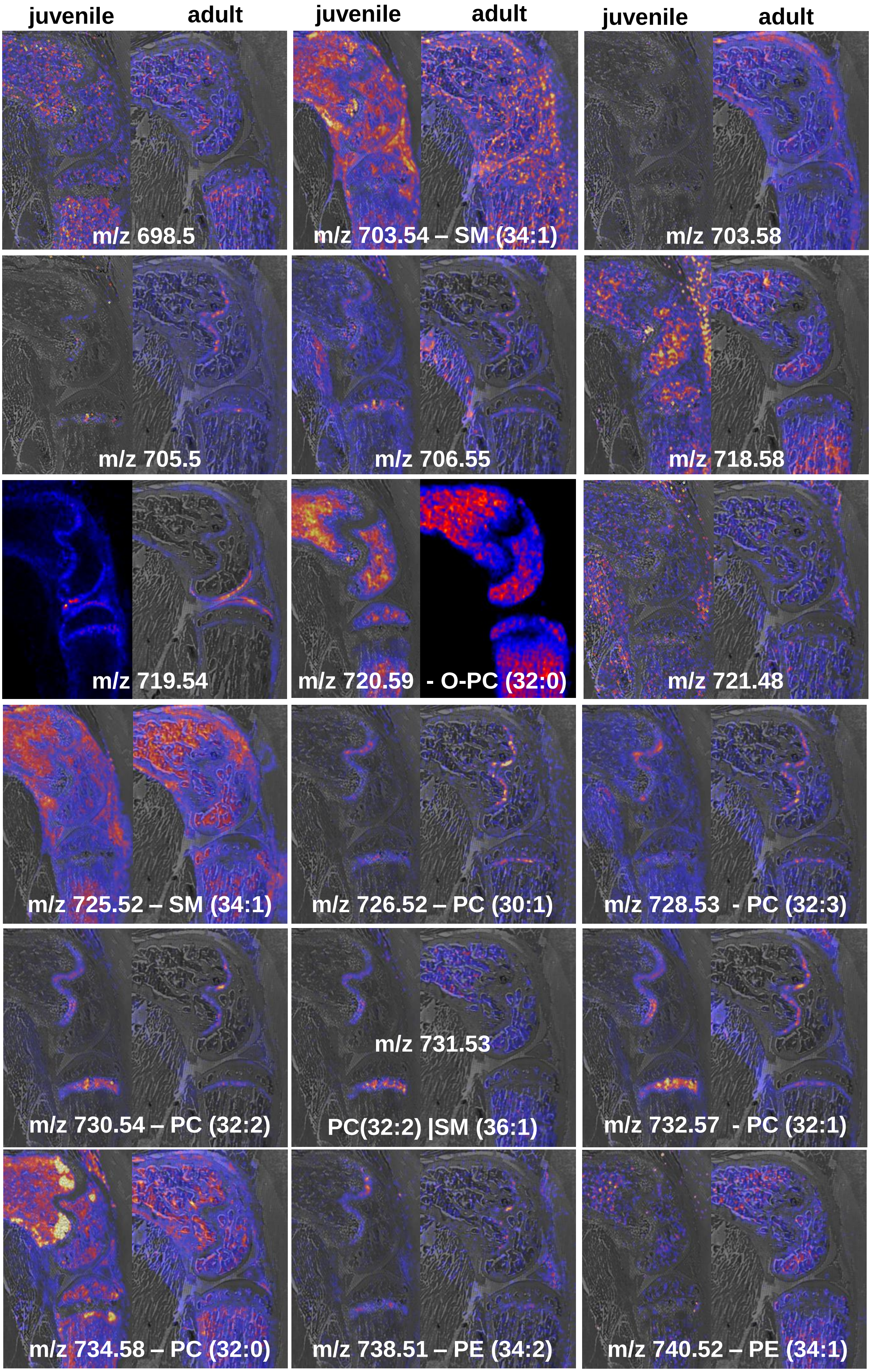
juvenile
adult
juvenile
adult
juvenile
adult
m/z 698.5
m/z 703.54 – SM (34:1)
m/z 703.58
m/z 705.5
m/z 706.55
m/z 718.58
m/z 719.54
m/z 720.59 - O-PC (32:0)
m/z 721.48
m/z 725.52 – SM (34:1)
m/z 726.52 – PC (30:1)
m/z 728.53 - PC (32:3)
m/z 730.54 – PC (32:2)
m/z 731.53
PC(32:2) |SM (36:1)
m/z 732.57 - PC (32:1)
m/z 734.58 – PC (32:0)
m/z 738.51 – PE (34:2)
m/z 740.52 – PE (34:1)

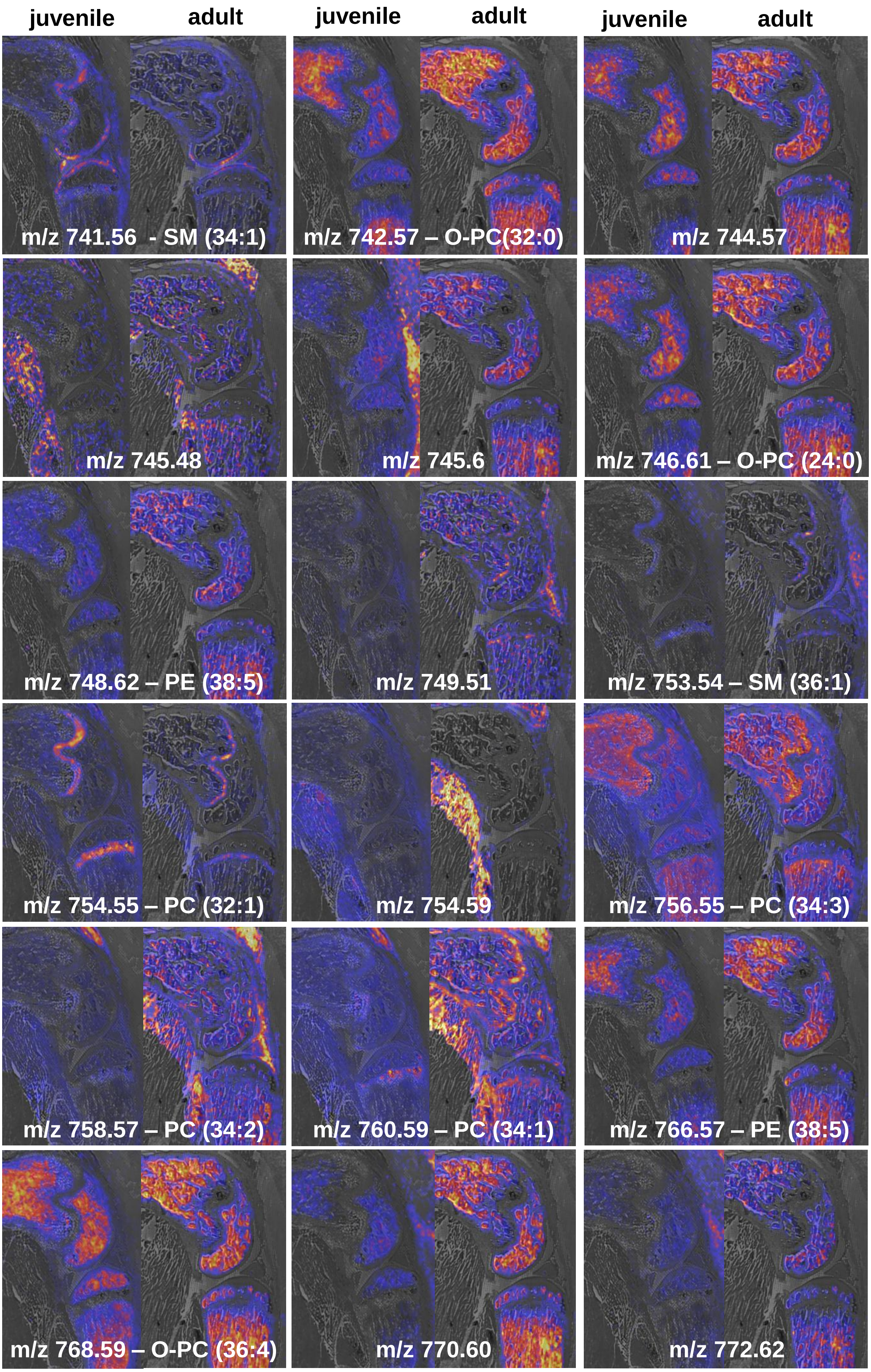
juvenile
adult
juvenile
adult
juvenile
adult
m/z 741.56 - SM (34:1)
m/z 742.57 – O-PC(32:0)
m/z 744.57
m/z 745.48
m/z 745.6
m/z 746.61 – O-PC (24:0)
m/z 748.62 – PE (38:5)
m/z 749.51
m/z 753.54 – SM (36:1)
m/z 754.55 – PC (32:1)
m/z 754.59
m/z 756.55 – PC (34:3)
m/z 758.57 – PC (34:2)
m/z 760.59 – PC (34:1)
m/z 766.57 – PE (38:5)
m/z 768.59 – O-PC (36:4)
m/z 770.60
m/z 772.62

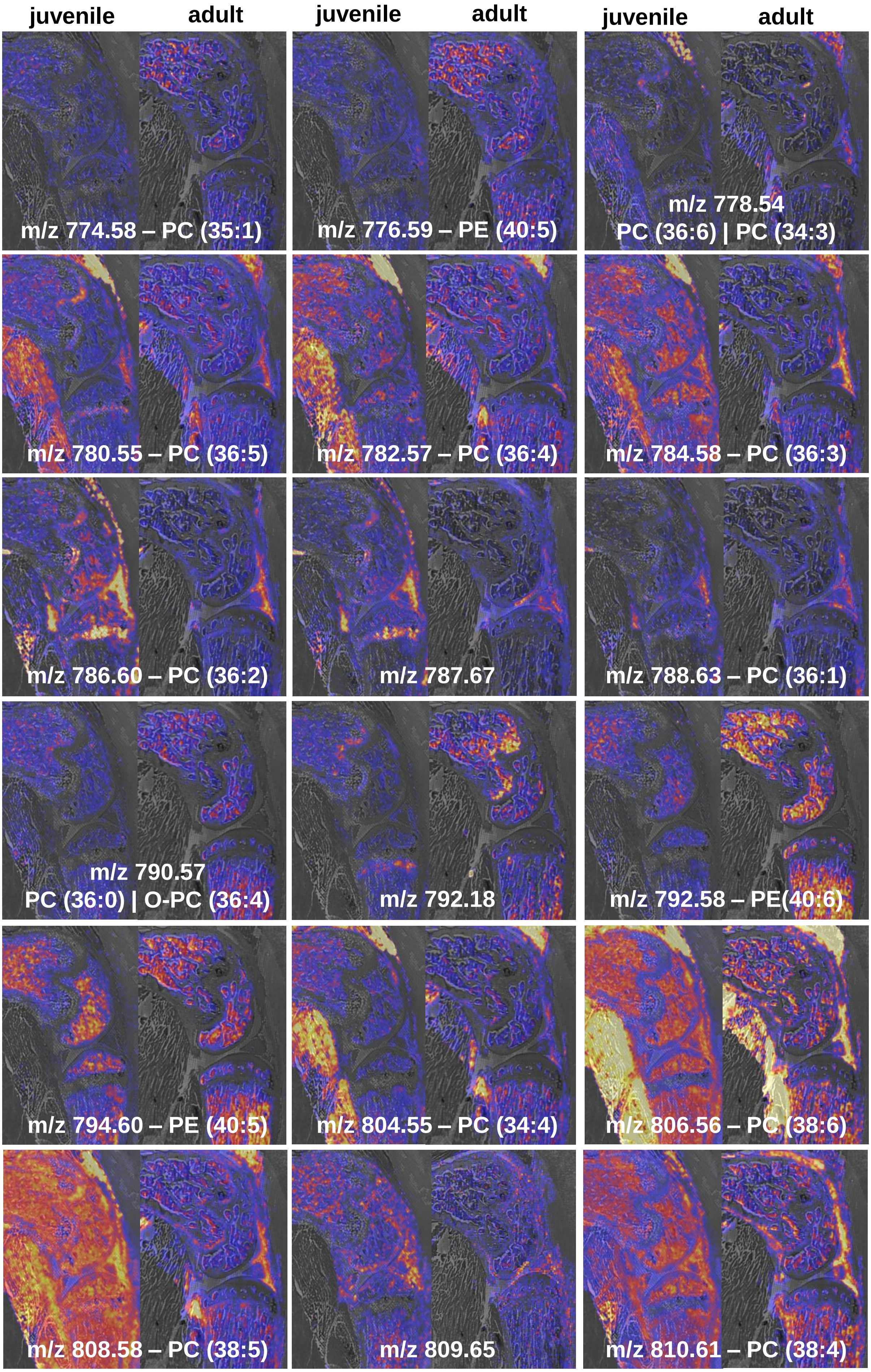
juvenile
adult
juvenile
adult
juvenile
adult
m/z 774.58 – PC (35:1)
m/z 776.59 – PE (40:5)
m/z 778.54
PC (36:6) | PC (34:3)
m/z 780.55 – PC (36:5)
m/z 782.57 – PC (36:4)
m/z 784.58 – PC (36:3)
m/z 786.60 – PC (36:2)
m/z 787.67
m/z 788.63 – PC (36:1)
m/z 790.57
PC (36:0) | O-PC (36:4)
m/z 792.18
m/z 792.58 – PE(40:6)
m/z 794.60 – PE (40:5)
m/z 804.55 – PC (34:4)
m/z 806.56 – PC (38:6)
m/z 808.58 – PC (38:5)
m/z 809.65
m/z 810.61 – PC (38:4)

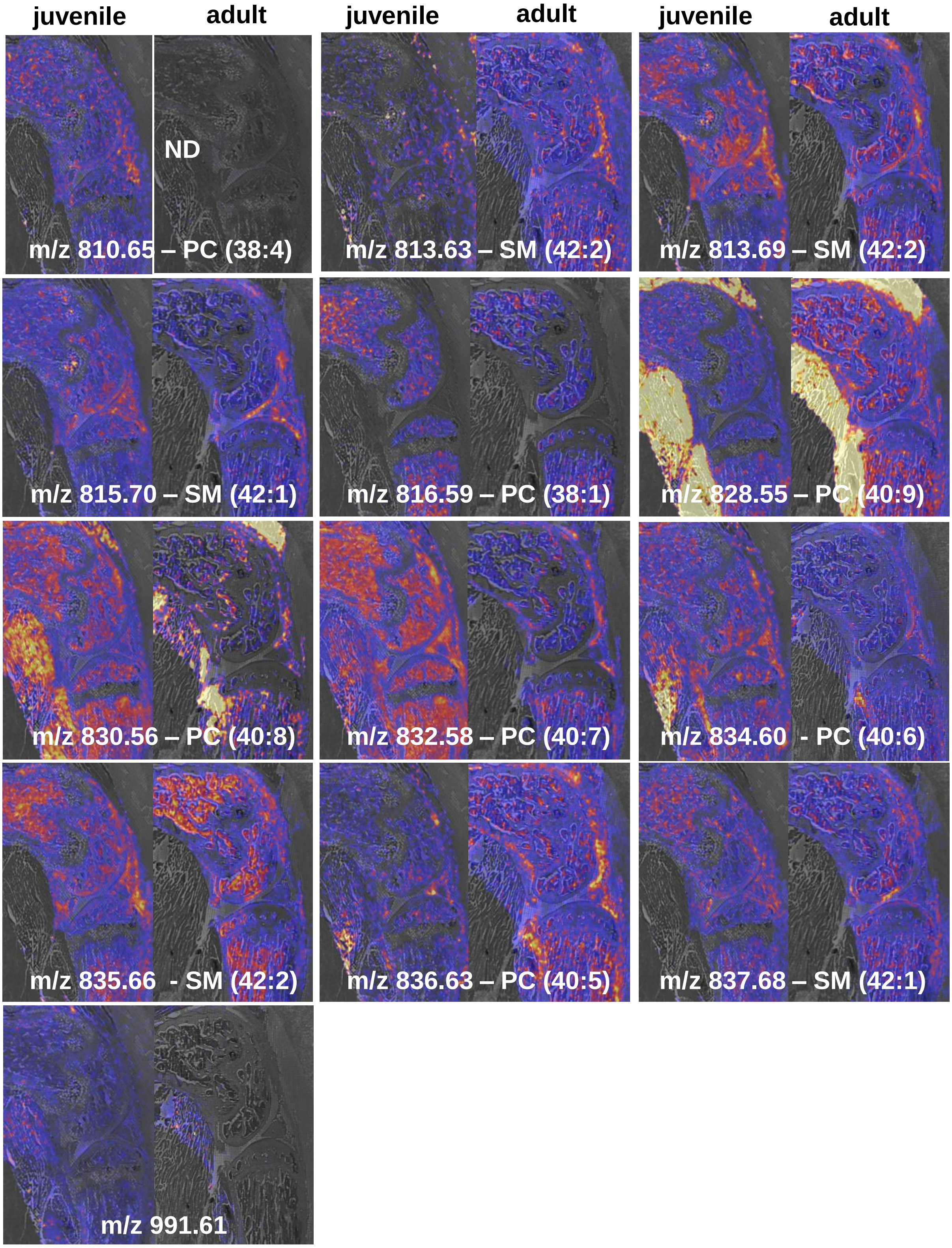
juvenile
adult
juvenile
adult
juvenile
adult
ND
m/z 810.65 – PC (38:4)
m/z 813.63 – SM (42:2)
m/z 813.69 – SM (42:2)
m/z 815.70 – SM (42:1)
m/z 816.59 – PC (38:1)
m/z 828.55 – PC (40:9)
m/z 830.56 – PC (40:8)
m/z 832.58 – PC (40:7)
m/z 834.60 - PC (40:6)
m/z 835.66 - SM (42:2)
m/z 836.63 – PC (40:5)
m/z 837.68 – SM (42:1)
m/z 991.61

Fig. S5

Lipid ion ID cards of manually annotated lipids. For each precursor ion of interest, an average LC-MS/MS spectrum was generated. LC-MS/MS was carried out on lipids extracted from tissue homogenate of the whole joint. The diagnostic peaks detected across the average HCD spectrum have been annotated. Deductions are described below each spectrum.

Abbreviations and nomenclature:
RT – retention time
PC – Phosphatidylcholine
PE – Phosphatidylethanolamie
SM – Sphingomyelin
FA – Fatty acid

Diagnostic ions were based on the LipidMatch annotation libraries.

m/z 482.33 | RT 96 | PC(15:0/0:0) | LysoPC

Positive Ion Mode MS2 spectrum

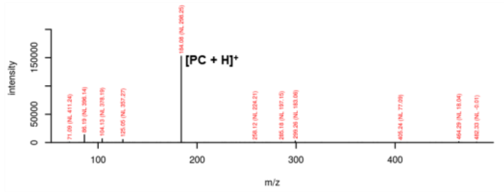

m/z 184 => PC

Negative Ion Mode MS2 spectrum

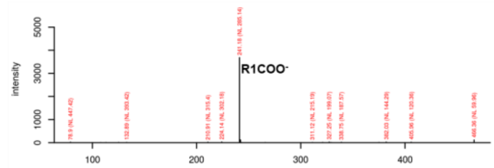

m/z 241 => FA 15:0; unlikely hit since FAs with uneven number of C atoms are uncommon in mammals; hit below more likely as more intense in the LCMS/MS and more likely to be detected in the less sensitive MALDI-IMS

m/z 482.35 | RT 183 | Plasmanyl-PC (O-16:0_0:0) | Plasmanyl-LysoPC

Positive Ion Mode MS2 spectrum

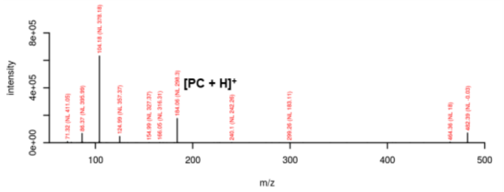

m/z 184 => PC

Negative Ion Mode MS2 spectrum

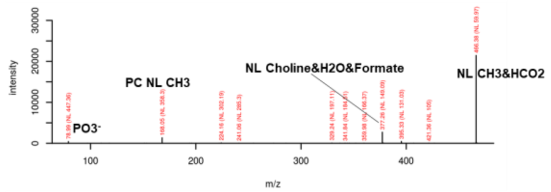

m/z 168 => PC | lack of R1COO- => plasmanyl-PC | precursor m/z 526.3 matches only Plasmanyl-PC (O-16:0/0:0)

m/z 494.3 | RT 94 | PC(16:1/0:0) | LysoPC

Positive Ion Mode MS2 spectrum

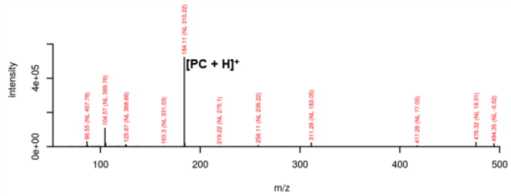

m/z 184 => PC

Negative Ion Mode MS2 spectrum

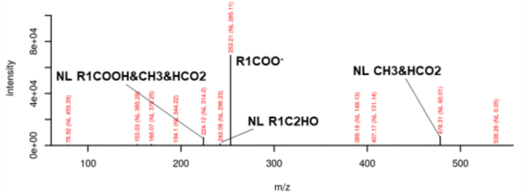

m/z 253 => FA 16:1

m/z 496.3 | RT 149 | PC (16:0/0:0) | LysoPC

Positive Ion Mode MS2 spectrum

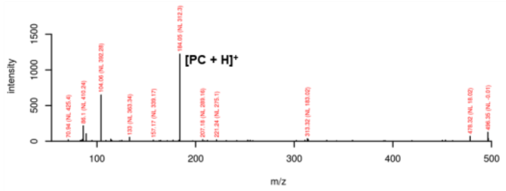

m/z 184 => PC

Negative Ion Mode MS2 spectrum

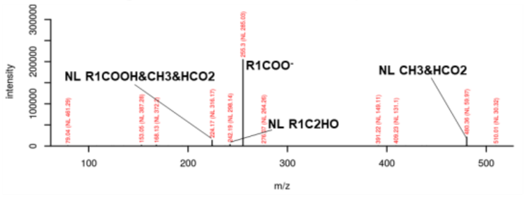

m/z 255 => FA 16:0

m/z 508.37 | RT 216 | Plasmanyl-PC(O-18:0/0:0) | Plasmanyl-LysoPC

Positive Ion Mode MS2 spectrum

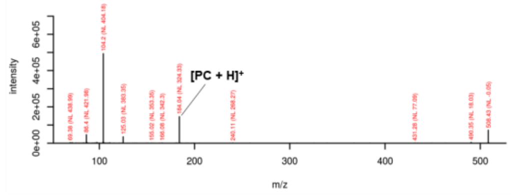

m/z 184 => PC

Negative Ion Mode MS2 spectrum

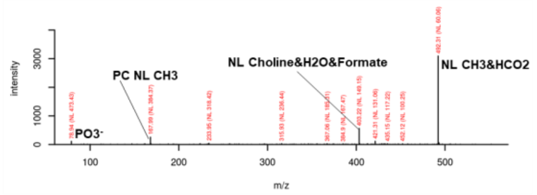

m/z 168 => PC | lack of R1COO- => plasmanyl-PC | precursor m/z 552.3 matches only Plasmanyl-PC (O-18:0/0:0)

m/z 518.3 | RT 85 | PC (18:3/0:0) | LysoPC

Positive Ion Mode MS2 spectrum

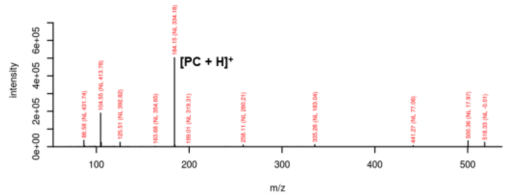

m/z 184 => PC

Negative Ion Mode MS2 spectrum

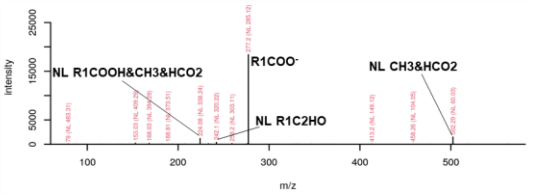

m/z 277 => FA 18:3

m/z 520.3 | RT 104 | PC(18:2/0:0) | LysoPC

Positive Ion Mode MS2 spectrum

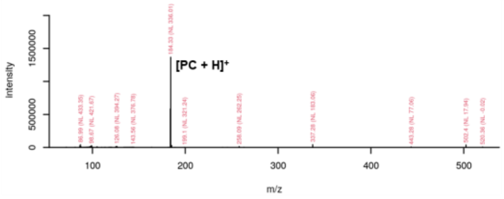

m/z 184 => PC

Negative Ion Mode MS2 spectrum

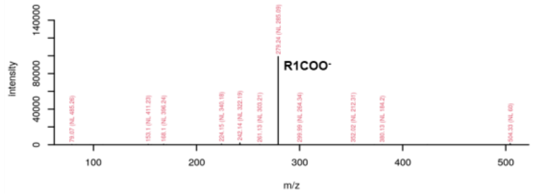

m/z 279.3 => FA 18:2

m/z 522.3 | RT 178 | PC (18:1/0:0) | LysoPC

Positive Ion Mode MS2 spectrum

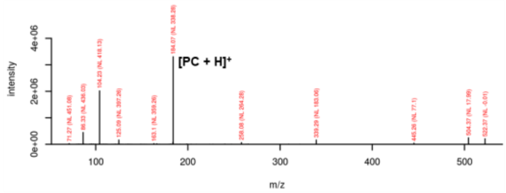

m/z 184 => PC

Negative Ion Mode MS2 spectrum

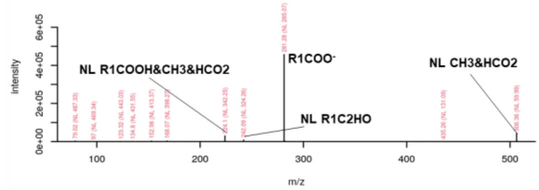

m/z 281 => FA 18:1

m/z 544.3 | RT 116 | PC(20:4/0:0) | LysoPC

Positive Ion Mode MS2 spectrum

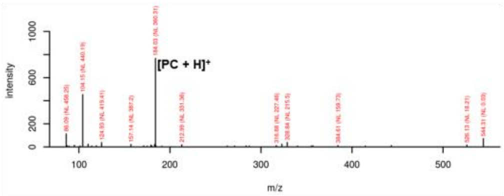

m/z 184 => PC

Negative Ion Mode MS2 spectrum

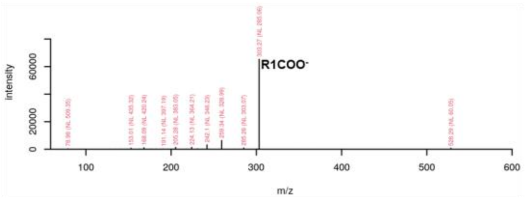

m/z 303.3 => FA 20:4

m/z 546.3 | RT 146 | PC(20:3/0:0) | LysoPC

Positive Ion Mode MS2 spectrum

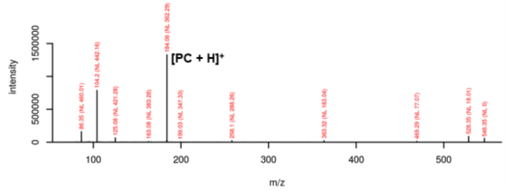

m/z 184 => PC

Negative Ion Mode MS2 spectrum

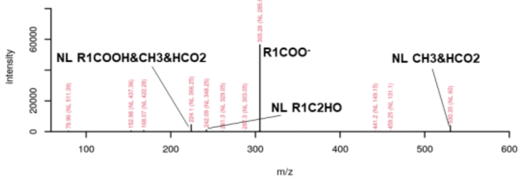

m/z 305.3 => FA 20:3

m/z 546.3 has the same distribution as m/z 524.3 -> PC(18:0/0:0) + Na is the more likely ID based on the putative hit for m/z 524.3

m/z 650.4 | RT 308 | PC(16:0_10:0) | PC

Positive Ion Mode MS2 spectrum

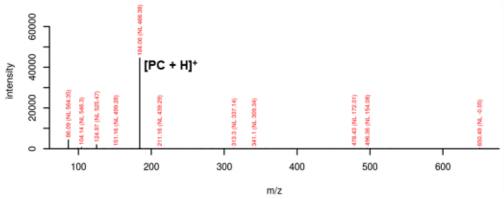

m/z 184 => PC

Negative Ion Mode MS2 spectrum

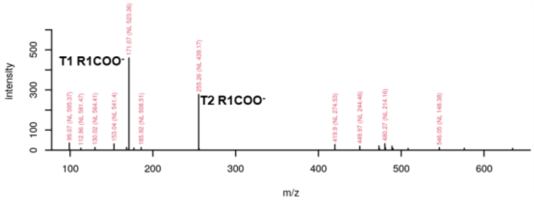

m/z 255.3 => FA 16:0
m/z 171.1 => FA 10:0

m/z 703.5 | RT 787 | SM(d18:1/16:0) | SM

Positive Ion Mode MS2 spectrum

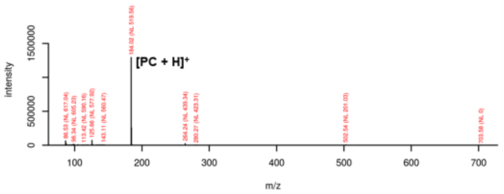

m/z 184 => PC headgroup
Precursor mass odd number => SM

Negative Ion Mode MS2 spectrum

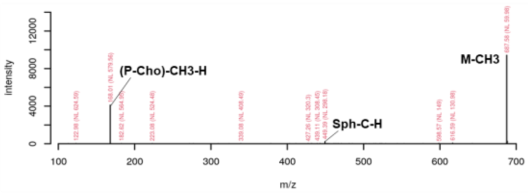

m/z 449.3 => d18:1

m/z 720.6 | RT 892 | Plasmanyl-PC(O-16:0/16:0) | Plasmanyl-PC

Positive Ion Mode MS2 spectrum

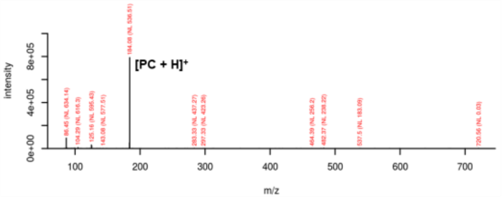

m/z 184 => PC

Negative Ion Mode MS2 spectrum

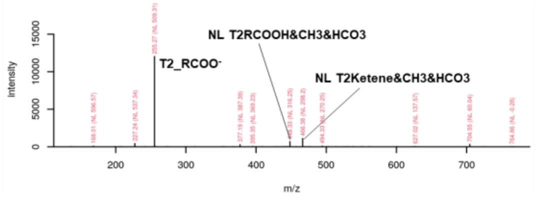

m/z 255.3 => FA 16:0; if PC it would also need a FA 12:1 fragment present => ether PC;
only plasmanyl-PC has matching precursor at m/z 764.6;
m/z 255.3, m/z 448.3, and m/z 466.4 diagnostic fragment ions of Plasmanyl-PC(O-16:0/16:0) are present

m/z 728.5 | RT 703 | PC(32:3) | PC

Positive Ion Mode MS2 spectrum

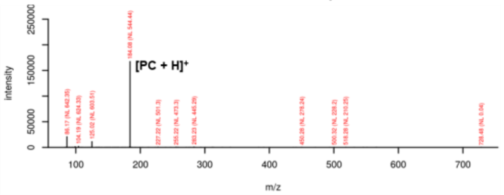

m/z 184 => PC

Negative Ion Mode MS2 spectrum

No matching negative mode spectrum => can't determine FA chain length

m/z 730.5 | RT 765 | PC(32:2) | PC

Positive Ion Mode MS2 spectrum

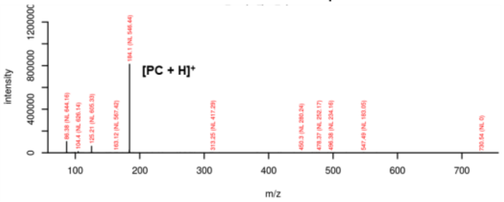

m/z 184 => PC

Negative Ion Mode MS2 spectrum

No matching negative mode spectrum => can't determine FA chain length

m/z 732.6 | RT 803 | PC(32:1) | PC

Positive Ion Mode MS2 spectrum

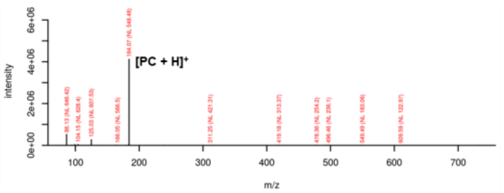

m/z 184 => PC

Negative Ion Mode MS2 spectrum

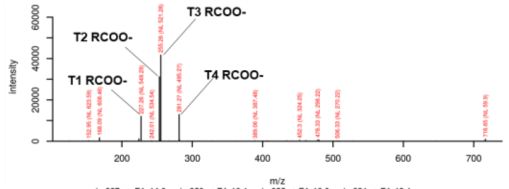

m/z 227 => FA 14:0; m/z 253 =>FA 16:1; m/z 255 =>FA 16:0; m/z 281 =>FA 18:1
Likely a mix of PC(16:0_16:1) and PC(14:0_18:1);
unlikely a cardiolipin since fragment's don't match the lipid match diagnostic fragments and precursor is too small

m/z 734.6 | RT 856 | PC(16:0/16:0) | PC

Positive Ion Mode MS2 spectrum

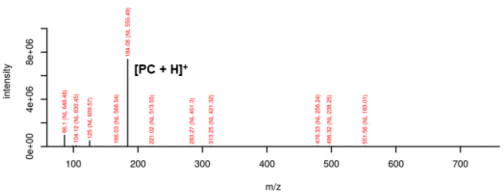

m/z 184 => PC

Negative Ion Mode MS2 spectrum

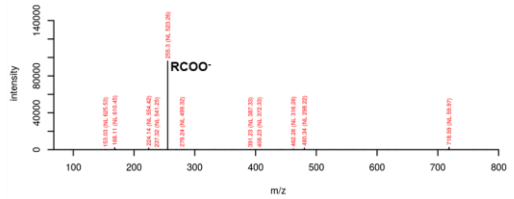

m/z 255 => FA 16:0; no other FA chain ions => 2 identical chains

m/z 746.6 | RT 893 | PC(O-18:1_16:0) | Plasmanyl-PC

Positive Ion Mode MS2 spectrum

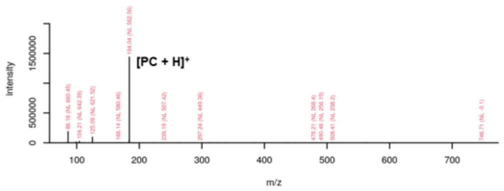

m/z 184 => PC

Negative Ion Mode MS2 spectrum

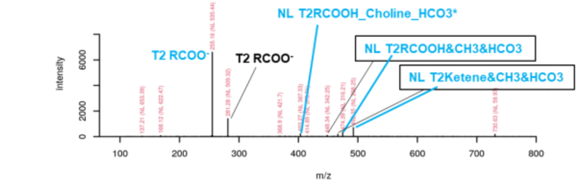

m/z 255 => FA 16:0; m/z 281 =>FA 18:1; does not match precursor m/z of a PC with these two chains but matches the m/z of a plasmanyl-PC PC(O-34:1). Diagnostic fragments are present for both Plasmanyl-PC(O-18:1/16:0) and Plasmanyl-PC(O-16:0/18:1), so the sn1 and sn2 positional isomers could not be separated.

m/z 754.55 | RT 744 | PC(14:0/20:4) | PC

Positive Ion Mode MS2 spectrum

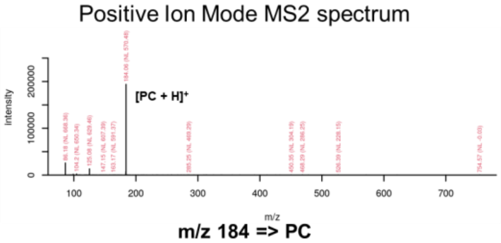

m/z 184 => PC

NB: m/z 754.55 has a similar spatial distribution to m/z 732.57; likely this is the M+Na of 732.57

Negative Ion Mode MS2 spectrum

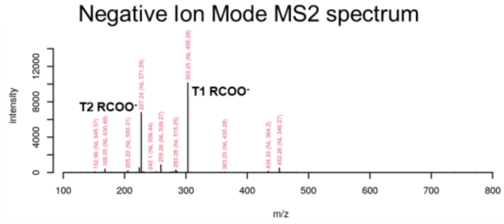

m/z 227 => FA 14:0; m/z 303 => FA 20:4

m/z 756.56 | RT 761 | PC(18:2_16:1) | PC

Positive Ion Mode MS2 spectrum

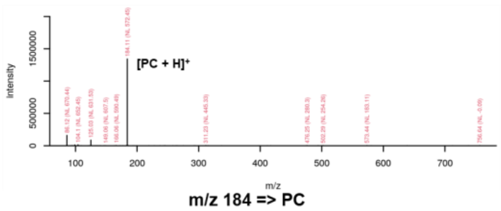

m/z 184 => PC

NB: m/z 756.56 has a similar spatial distribution to m/z 734.57; likely this is the M+Na of 734.57

Negative Ion Mode MS2 spectrum

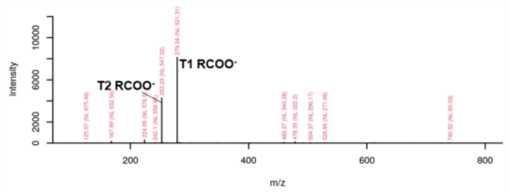

m/z 253 => FA 16:1; m/z 279 =>FA 18:2

m/z 758.57 | RT 813 | PC(34:2) | PC

Positive Ion Mode MS2 spectrum

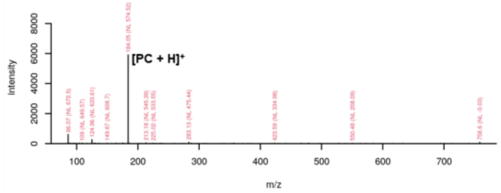

m/z 184 => PC

Negative Ion Mode MS2 spectrum

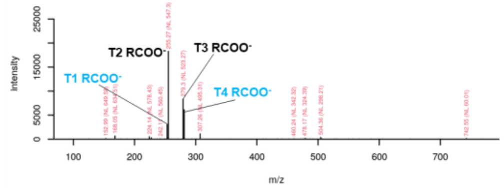

m/z 253 => FA 16:1; m/z 255 => FA 16:0; m/z 279 => FA 18:2; m/z 281 => FA 18:1
Likely a mix of PC(16:0_18:2) and PC(16:1_18:1)

m/z 760.6 | RT 861 | PC(16:0_18:1) | PC

Positive Ion Mode MS2 spectrum

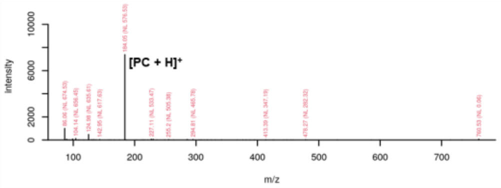

m/z 184 => PC

Negative Ion Mode MS2 spectrum

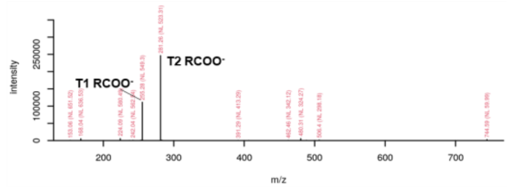

m/z 255 => FA 16:0; m/z 281 =>FA 18:1

m/z 766.58 | RT 826 | PE(38:5) | PE

Positive Ion Mode MS2 spectrum

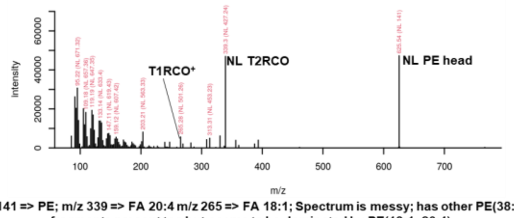

NL 141 => PE; m/z 339 => FA 20:4 m/z 265 => FA 18:1; Spectrum is messy; has other PE(38:5) fragments present too but seems to be dominated by PE(18:1_20:4)

Negative Ion Mode MS2 spectrum [M-H]-

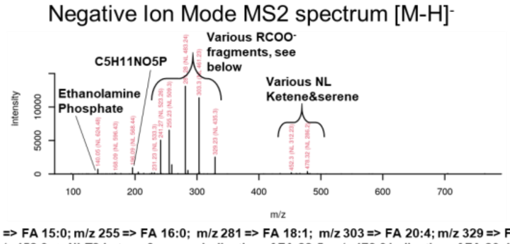

m/z 241 => FA 15:0; m/z 255 => FA 16:0; m/z 281 => FA 18:1; m/z 303 => FA 20:4; m/z 329 => FA22:5
m/z 452.3 => NLT2 ketene & serene indicative of FA 22:5; m/z 478.3 indicative of FA 20:4

m/z 768.59 | RT 847 | PC(O-16:0/20:4) | PC

Positive Ion Mode MS2 spectrum

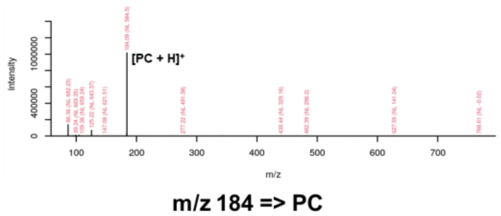

m/z 184 => PC

Negative Ion Mode MS2 spectrum

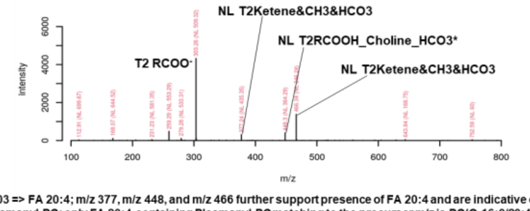

m/z 303 => FA 20:4; m/z 377, m/z 448, and m/z 466 further support presence of FA 20:4 and are indicative of a Plasmanyl-PC; only FA 20:4-containing Plasmanyl-PC matching to the precursor m/z is PC(O-16:0/20:4)

m/z 774.59 | RT 887 | PC(35:1) | PC

Positive Ion Mode MS2 spectrum

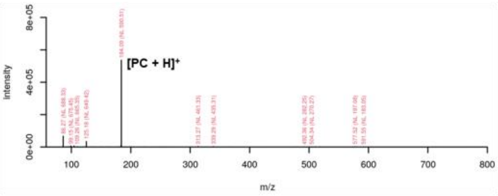

m/z 184 => PC

Negative Ion Mode MS2 spectrum

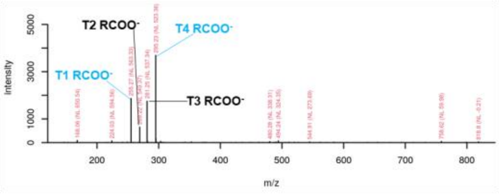

m/z 255 => FA 16:0; m/z 269 => FA 17:0; m/z 281 => FA 18:1; m/z 295 => FA 19:1
Likely a mix of PC(17:0_18:1) and PC(16:0_19:1)

m/z 780.56 | RT 731 and RT 771 | PC(36:5) | PC

Positive Ion Mode MS2 spectrum RT 731

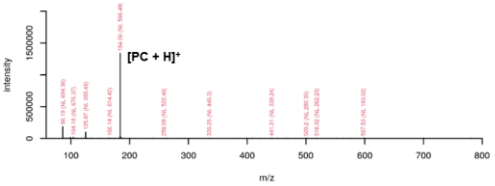

m/z 184 => PC

NB: Another ion with near identical m/z was detected at RT 771, also a PC

Negative Ion Mode MS2 spectrum RT 732

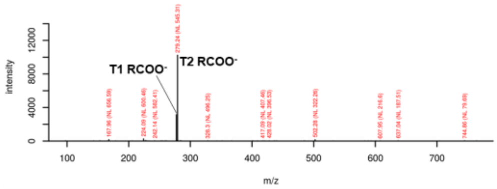

m/z 277 => FA 18:3; m/z 279 =>FA 18:2; PC(18:2_18:3)

NB: Another ion with near identical m/z was detected at RT 771, also a PC(36:5), so this ion was assigned as PC(36:5) for the MALDI analyses

m/z 782.57 | RT 775 and RT 810 | PC(36:4) | PC

Positive Ion Mode MS2 spectrum RT 775

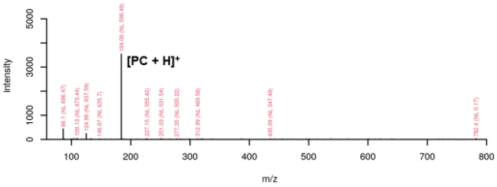

m/z 184 => PC

NB: Another ion with near identical m/z was detected at RT 810, also a PC

Negative Ion Mode MS2 spectrum RT 776

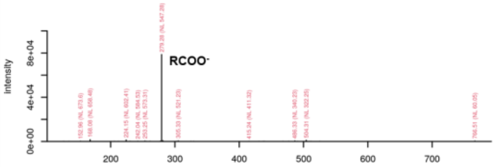

m/z 279 =>FA 18:2; no other RCOO- => identical sn1 and sn2 chains

NB: Another ion with near identical m/z was detected at RT 809, also a PC(36:4), so this ion was assigned as PC(36:4) for the MALDI analyses

m/z 784.59 | RT 833 | PC(36:3) | PC

Positive Ion Mode MS2 spectrum

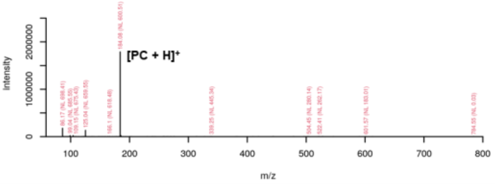

m/z 184 => PC

Negative Ion Mode MS2 spectrum

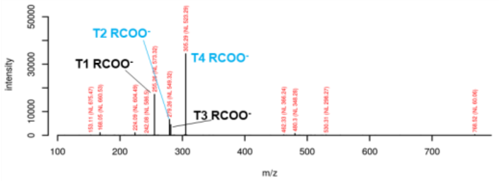

m/z 255 => FA 16:0; m/z 279 => FA 18:2; m/z 281 =>FA 18:1; m/z 305 => FA20:3
=> Mix of PC(16:0_20:3) and PC(18:1_18:2)

m/z 786.6 | RT 875 | PC(36:2) | PC

Positive Ion Mode MS2 spectrum

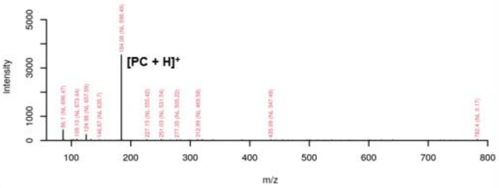

m/z 184 => PC

Negative Ion Mode MS2 spectrum

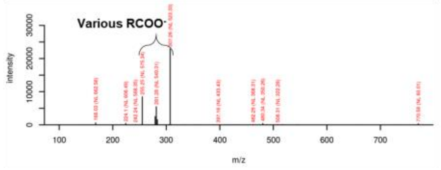

m/z 255 => FA 16:0; m/z 279 =>FA 18:2; m/z 281 => FA 18:1; m/z 283 => FA 18:0; m/z 307 => FA 20:2
=> Mix of PC(16:0_20:2), PC(18:0_18:2), and PC(18:1/18:1) => assign PC(36:2)

m/z 788.63 | RT 913 | PC(36:1) | PC

Positive Ion Mode MS2 spectrum

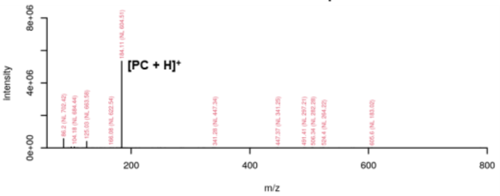

m/z 184 => PC; precursor m/z matches PC(36:1)

Negative Ion Mode MS2 spectrum

No matching negative mode spectrum => can't determine FA chain length

m/z 790.58 | RT 875 | PC(36:0) | PC

Positive Ion Mode MS2 spectrum

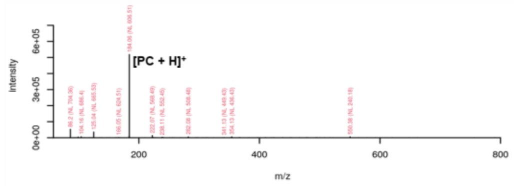

m/z 184 => PC (36:0)
NB: Could also be the [M+Na]⁺ of m/z 768.59 – PC(O-16:0/20:4)

Negative Ion Mode MS2 spectrum

No matching negative mode spectrum => can't determine FA chain length

m/z 792.59 | RT 865 | PE(18:0_22:6) | PE

Positive Ion Mode MS2 spectrum

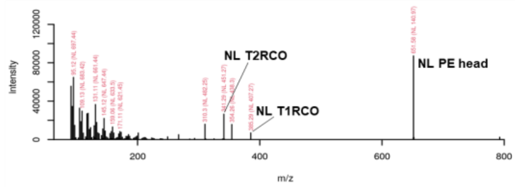

NL 141 => PE; m/z 341 => FA 18:0 m/z 385 => FA 22:6)
310 and 354 did not have a diagnostic match in the lipid match library

Negative Ion Mode MS2 spectrum

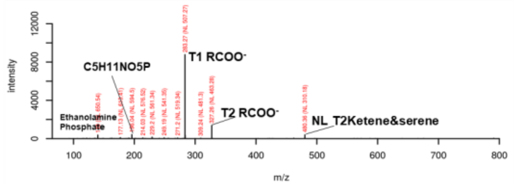

m/z 283 => FA 18:0; m/z 327 => FA 22:6

m/z 794.61 | RT 875 | PE(40:5) | PE

Positive Ion Mode MS2 spectrum

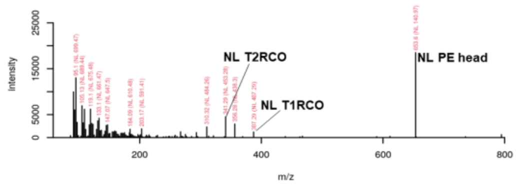

NL 141 => PE; m/z 341 => FA 18:0 m/z 387 => FA 22:5
310 and 354 did not have a diagnostic match in the lipid match library

Negative Ion Mode MS2 spectrum

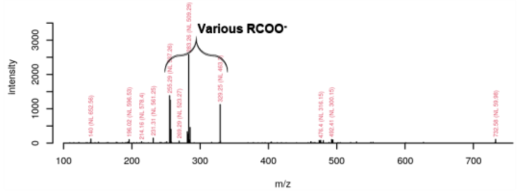

Due to multiple FA chains present in negative mode, the ID was set to PE(40:5); based on positive mode it seems likely to be PE(18:0_22:5)

m/z 808.59 | RT 813 | PC(38:5) | PC

Positive Ion Mode MS2 spectrum

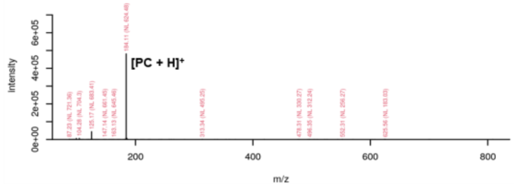

m/z 184 => PC

Negative Ion Mode MS2 spectrum

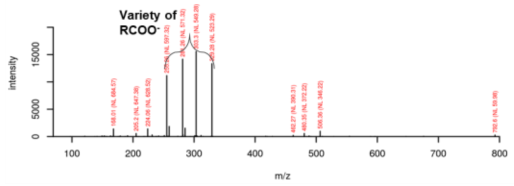

Due to multiple FA chains present, the ID was set to PC(38:5)

m/z 810.62 | RT 837 | PC(38:4) | PC

Positive Ion Mode MS2 spectrum

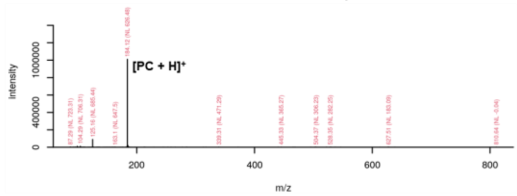

m/z 184 => PC
NB: multiple ions in LCMS/MS at RT838, RT849, and RT 869, all PCs due to 184 fragment presence

Negative Ion Mode MS2 spectrum

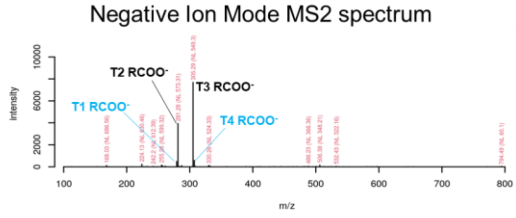

m/z 279 => FA 18:2; m/z 281 => FA 18:1; m/z 305 => FA 20:3; m/z 307 => FA20:2
Mix of predominantly PC(18:1_20:3) and some PC(18:2_20:2) => PC(38:4)

m/z 813.69 | RT 953 | SM(d18:1/24:1) | SM

Positive Ion Mode MS2 spectrum

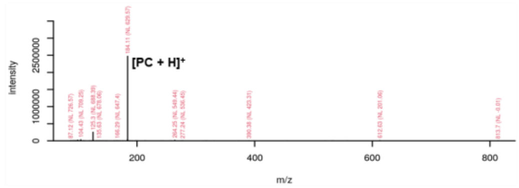

m/z 184 with odd precursor m/z => SM

Negative Ion Mode MS2 spectrum

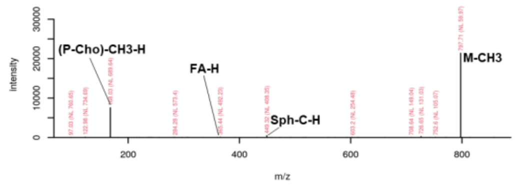

m/z 365 => FA 24:1; m/z 449=> d18:1

m/z 815.7| RT 1005 | SM(42:1) | SM

Positive Ion Mode MS2 spectrum

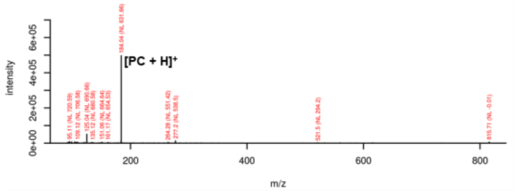

m/z 184 with odd precursor m/z => SM

Negative Ion Mode MS2 spectrum

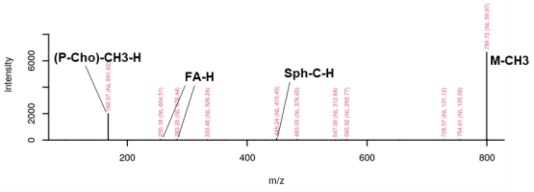

m/z 255 => FA 16:0; m/z 283 => FA 18:0; m/z 449 => d18:1; likely a mix of multiple isobaric SMs, including SM(d18:1/24:0), SM(d24:1/18:0), and SM(d26:1/16:0) => assign as SM(42:1)

NB in neg mode another ion with similar m/z and RT 970 was fragmented, also a SM, where m/z 255 was the most intense FA ion, indicative of FA 16:0

m/z 816.6 | RT 674 and RT654 | PC(38:1) | PC

Positive Ion Mode MS2 spectrum RT 674

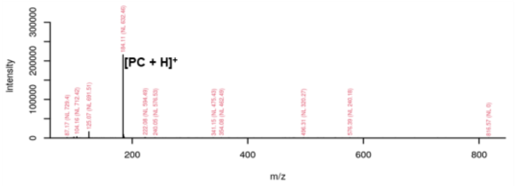

m/z 184 => PC

NB: Another ion with near identical m/z was detected at RT 654, also a PC

Negative Ion Mode MS2 spectrum RT 675

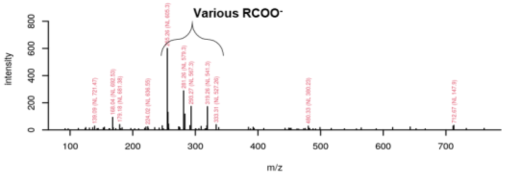

Messy spectrum with multiple RCOO- FA fragments; assigned as PC (38:1)

NB: Another ion with near identical m/z was detected at RT 660, also PC(38:1)

m/z 504.3 | no LC-MS/MS match | 482.33 + Na^+ AND 522.3 – H2O + H^+

The spatial distribution of the ion in juvenile mice was indicative of two lipids resolved in the ion mobility phase. In adult mouse data, the 504.3 ion was present in marrow predominantly, in juvenile data two ions with m/z close to 504.3 but different distribution were detected, indicative of two separate lipids with similar m/z.

The already identified m/z 482.33 and m/z 522.3 are protonated lipids [M+ H^+], with their sodiated and dehydrated adducts respectively expected at m/z 504.3. Given that m/z 482.33 is enriched in marrow and m/z 522.3 is elevated in the growth plate, this would explain the detection of two ions very close to m/z 504.3 in the juvenile mouse.

Fig. S6

A **PCA hypertrophic zone ROIs**

B **PCA proliferating zone ROIs**

C **PCA resting zone ROIs**

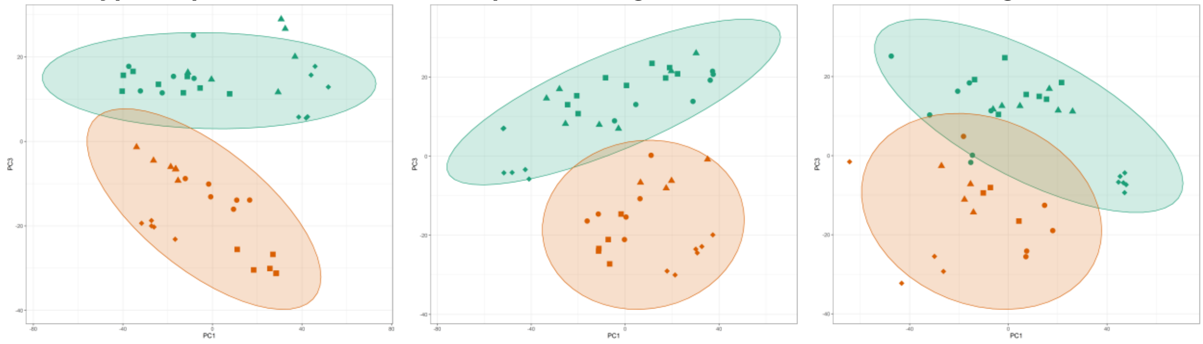

WT vs KO PCA scores plots by zone**.**
